# Perceived benefits and disadvantages for healthcare professionals when implementing digital health technologies in breast cancer care: A systematic review

**DOI:** 10.1177/20552076251404497

**Published:** 2025-12-04

**Authors:** Julia Wendel, Anna-Lena Hofmann, Jonas Widmann, Achim Wöckel, Peter Heuschmann, Jens-Peter Reese

**Affiliations:** 1 Institute for Clinical Epidemiology and Biometry, 9190 University of Würzburg, Würzburg, Germany; 2 Institute for Medical Data Science, University Hospital Würzburg (UKW), Würzburg, Germany; 3 Department of Gynecology and Obstetrics, University Hospital Würzburg (UKW), Würzburg, Germany; 4 Clinical Trial Centre, University Hospital Würzburg (UKW), Würzburg, Germany; 5 Technische Hochschule Mittelhessen, University of Applied Sciences, Gießen, Germany

**Keywords:** Digital health technologies, review, breast cancer, healthcare professional, implementation

## Abstract

**Background:**

Digital health technologies (DHTs) are increasingly being used in healthcare, including breast cancer care and aftercare, to improve patient-related outcomes and efficiently organise care processes. This systematic review aims to synthesise existing evidence on perceived advantages and disadvantages of adopting DHTs from the perspective of healthcare professionals (HCPs).

**Methods:**

A comprehensive literature search was conducted across PubMed, IEEE Xplore and PsycInfo using a peer-reviewed search strategy. Studies addressing advantages and disadvantages of DHTs in breast cancer care with a focus on HCPs’ perspectives were included. A thematic synthesis approach was employed to extract and categorise qualitative and quantitative findings. The quality assessment of the included studies was carried out using Mixed-Methods Appraisal Tool and Critical Appraisal Skills Programme Checklist. The protocol was registered on PROSPERO (CRD42024508808).

**Results:**

The database search retrieved 657 records, of which 21 reports were included in the final analysis. HCPs mentioned several advantages of DHTs, including improved patient care as well as support for clinical decision-making. Disadvantages included concerns about clinical accuracy and increased complexity in clinical workflows. Factors that facilitated the adoption of DHTs involved user-centred design and the perception of improved care quality. As barriers a lack of technical infrastructure and doubts about clinical benefits were mentioned.

**Conclusion:**

Although HCPs recognise the potential of DHTs to improve the quality of care, technical and organisational barriers can limit widespread adoption. Involving HCPs in the development and implementation of DHTs can support the sustainable integration of DHTs into breast cancer care.

## Background

Digital health technologies (DHTs) such as wearables, decision support systems and telemedicine have the potential to reduce direct and indirect costs in healthcare as well as improve the quality of care.^1^ Various international and national efforts have been made to strengthen healthcare systems through the use of DHTs. For example, the World Health Organization has endorsed the Global Strategy for Digital Health 2020–2025,^2^ which includes promoting the appropriate use of DHTs. Since summer 2021, physicians in Germany have been permitted to prescribe certified health applications to individuals covered by statutory health insurance. However, a survey conducted among German general practitioners, physicians and psychotherapists shows that only a third of them intend to prescribe mobile health (mHealth) apps, despite regulatory support.^3^ Common barriers to adoption include insufficient technical infrastructure, psychological reservations and concerns regarding workload and workflow integration. Enabling factors include training, usefulness and willingness to use from the perspective of healthcare professionals (HCPs) as well as incentivising multiple stakeholders.^4^

A variety of DHTs are used in breast cancer prevention, early detection, treatment coordination and post-treatment support.^5–7^ Existing reviews show encouraging results for DHTs in breast cancer care, particularly with regards to patient self-efficacy and coping skills,^8,9^ while others show no clear benefit of psychological mHealth interventions addressing breast cancer survivors’ mental health.^10^ While much of the current research in the field of breast cancer focuses on patient outcomes, less attention has been paid to the perspectives of HCPs,^11^ who play a crucial role in the adoption and implementation of these technologies. HCPs are often the primary point of contact for patients, providing guidance on the use of digital tools and integrating them into clinical workflows. Although a general review exists on barriers and facilitators of DHT adoption among HCPs,^4^ to our knowledge there is currently no systematic review specific to breast cancer care. The aim of this systematic review is to identify previously published scientific studies to analyse perceived advantages and disadvantages for HCPs in adopting DHTs and provide insight into the barriers and facilitators for the adoption of DHTs for HCPs.

## Methods

The review protocol was registered on PROSPERO (CRD42024508808). The results are summarised according to the Preferred Reporting Items for Systematic Reviews and Meta-Analyses (PRISMA) statement.^12^

### Search strategy

A search was conducted using pre-defined PICO criteria (population, intervention, comparison, outcome), see table 1, to investigate the perceived advantages and disadvantages for HCPs when adopting DHTs in breast cancer care.

**Table 1.** PICO criteria for the systematic literature search.

Population	Physicians or nurses in breast cancer care
Intervention	Digital health technologies
Comparison	Usual care/none *Not only RCTs are considered, therefore non-traditional control groups (e.g. pre-/post-design) are also eligible*
Outcome	Perceived benefits and disadvantages when implementing digital health technologies (Q1) as well as barriers and facilitators for the adoption of digital health technologies (Q2)

RCTs: randomised controlled trials.

Original research articles were included if they described the perspective of HCPs in the context of DHTs. Following the definition of Murray et al.,^13^ DHTs were defined as interventions delivered via digital technologies. In general, these digital interventions have an active component, such as informative and educational content or opportunities for interaction between patients or between patients and HCPs.^13^ Regarding the comparator, non-traditional control groups (e.g. pre-/post-design) or no comparators were also eligible, since not only randomised controlled studies were included. Articles were eligible if they described perceived benefits and disadvantages for HCPs when adopting DHTs in breast cancer care or barriers and facilitators to implementation as an outcome of the study. We excluded studies if only patient-related outcomes were reported. No restrictions were applied to study design. However, editorials, commentaries, study protocols, reviews, meta-analyses and guidelines were excluded. Given the socio-technical complexity of DHTs, the inclusion of qualitative evidence enables a deeper understanding of contextual factors, HCPs attitudes and perceived usability. These insights complement quantitative findings and are critical for implementation-oriented synthesis.

The electronic databases MEDLINE (via PubMed), PsycInfo (via ProQuest) and IEEE (via IEEE Xplore) were searched to identify relevant articles. No restrictions regarding date of publication or language were applied. The search of the three databases was conducted on 2024/02/28 (=literature search end date). Additionally, reference lists of included articles were screened manually for further eligible studies. Detailed information on the search strategy is provided in Table S1 of the Supplemental material I.

### Study selection

The results of the database search were exported into the Endnote Reference Manager and the Rayyan.ai software tool for systematic reviews.^14^ Duplicates were removed automatically and verified manually. The titles and abstracts of all articles were provided to two independent reviewers. The reviewers (JuWe, A-LH, JoWi) assessed whether the article met the inclusion or exclusion criteria at the title/abstract level. In the event of disagreements between the reviewers that could not be resolved after joint discussion, a third reviewer was consulted in order to reach a consensus.

### Data extraction and synthesis of results

Owing to high levels of heterogeneity between study types, interventions and outcome measurements, quantitative and qualitative results were analysed through a data-based convergent synthesis.^15^ In order to analyse and synthesise the data, the approach of Thomas and Harden^16^ was followed. This initially involves free line-by-line coding by two independent researchers and subsequent abstraction into descriptive themes and analytical categories. In quantitative studies, the relevant results sections were used to extract thematic content. The analysis was carried out using MAXQDA 24.^17^

### Risk of bias assessment for individual studies

Risk of bias was evaluated by two independent researchers. Disagreements were resolved through discussion or, if necessary, adjudicated by a third reviewer. For quantitative descriptive and mixed-methods studies the Mixed-Methods Appraisal Tool^18^ (MMAT) was used to assess individual risk of bias. For qualitative studies the Critical Appraisal Skills Programme (CASP) checklist^19^ was applied. The selection of the assessment tool was based on the methodology used to measure the outcome relevant to the review. The MMAT was used to assess the methodological quality of quantitative descriptive and mixed-methods studies and the CASP checklist was applied to qualitative studies.

## Results

### Study selection

**Figure 1.**
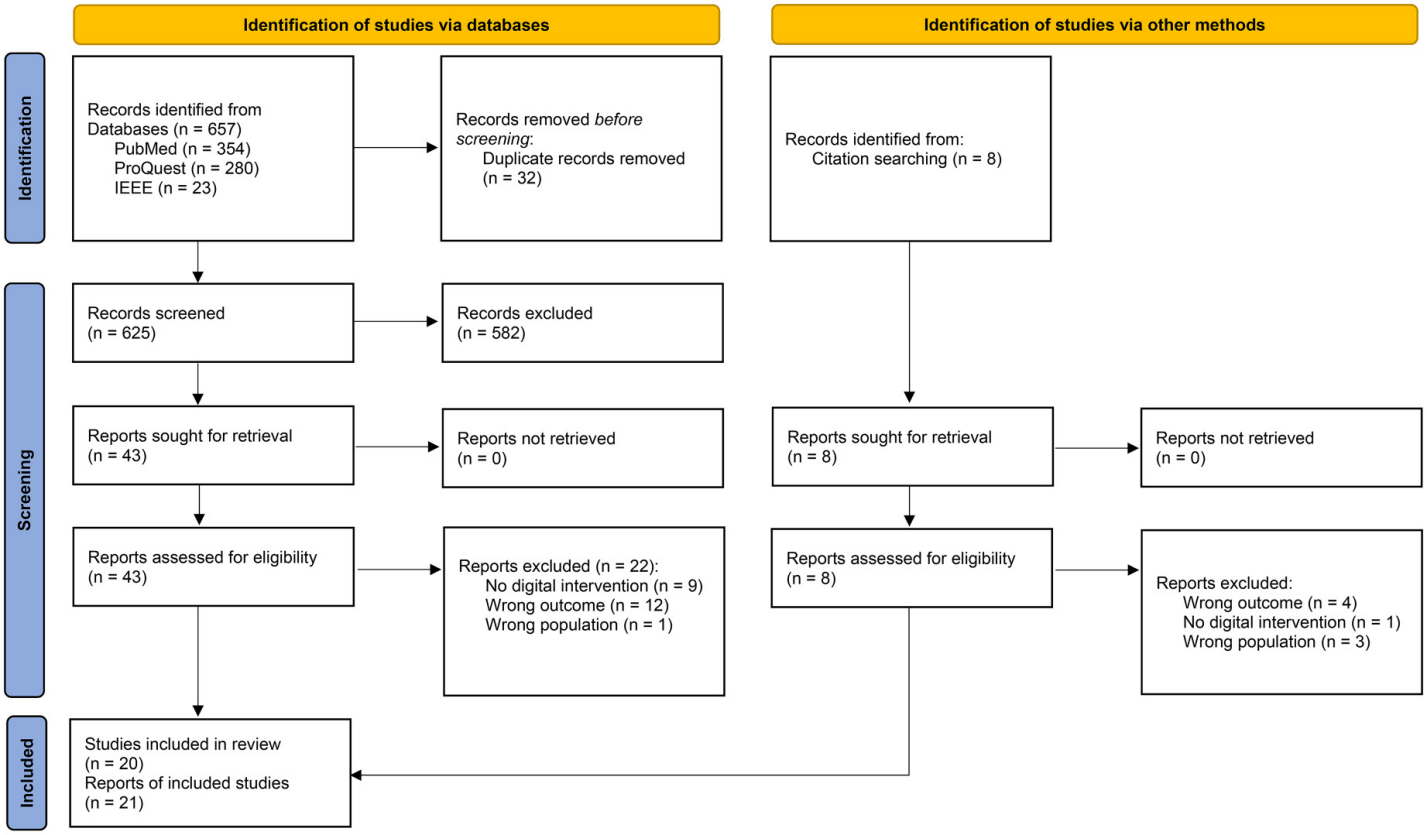
PRISMA flowchart for study selection. *Source*: Page MJ, et al.^12^ BMJ 2021;372:n71. doi: 10.1136/bmj.n71. This work is licensed under CC BY 4.0. To view a copy of this license, visit https://creativecommons.org/licenses/by/4.0/. PRISMA: Preferred Reporting Items for Systematic Reviews and Meta-Analyses.

The database search retrieved 657 records, of which 32 duplicates were removed. After screening titles and abstracts, 582 records were excluded, followed by the exclusion of 21 records during full-text review. After screening all cited sources from the included articles, eight full texts were retrieved, all of which were excluded. The excluded studies and the reasons for exclusion are provided in detail in Table S2 of the Supplemental material I. Consequently, 21 reports of 20 studies were included in the final analysis.^20–40^ The study selection process, following the PRISMA guidelines, is illustrated in Figure 1. The PRISMA checklist is provided in the Supplemental material IV.

### Study characteristics

The studies were mainly conducted in the USA^25–27^^,32,34–36,38–40^ and the UK,^21–23^^,30,31,37^ but also in other countries (the Netherlands,^33,41^ Sweden,^28^ France,^20^ Germany^29^) representing different healthcare systems. With regards to the assessment of the outcome relevant to the review, most studies employed quantitative methodologies,^20,26,27,29^^32–34^^,36,38,40^ complemented by mixed-methods^21,28,30,35,37,41^ and qualitative^22,23,25,31,39^ studies. The quantitative studies were mainly cross-sectional studies in the form of surveys,^29,32,34,36,38^ followed by two discrete choice experiments,^26,27^ a routine data analysis in the intervention arm of a randomised controlled trial,^20^ a process evaluation within a pre- and post-implementation trial^33^ as well as a retrospective observational study.^40^ In most of the mixed-methods studies, the qualitative data was collected in a verbal form, using interviews,^21,30,35^ focus groups^41^ and ad hoc verbal feedback.^37^ Two studies combined this qualitative data with quantitative data from questionnaires,^30,41^ one with quantitative usage data.^21^ One study^28^ collected qualitative and quantitative data within an online survey. Two studies used various data sources (observational clinical field-testing, including brief written feedback, direct observation, documentation, ad hoc verbal feedback^37^ or usage information from the website combined with close-ended feedback forms and open-ended interviews).^35^ Three qualitative studies used semi-structured interviews,^22,23,39^ one as informal feedback^25^ and one was an interpretive field study including interviews, observations, documentation analysis and data from informal discussions.^31^ Regarding the setting in breast cancer care, most studies focused on breast cancer treatment^21–23^^,29–31,33,35–37,39,41^ with one also addressing aftercare.^37^ Five studies were in the setting of primary care^34^ and breast cancer screening,^26–28^^,32^ two studies in the setting of breast cancer aftercare.^25,38^ Two studies did not specify the setting, referring generally to breast cancer management^20^ or hospital patient encounters (unspecified).^40^ The studies were published between 2008 and 2023 (2008–2010,^23,30,32^ 2011–2020,^20,22,25,29,31,34,35,37,39,41^ 2021–2023^21^^26–28^^,33,36,38,40^). The same intervention is reported in two included publications, initially as a feasibility trial^35^ and subsequently as a quality improvement study.^39^ Table S3 of the Supplemental material I describes the characteristics of the included studies in detail.

### Characteristics of DHTs

The DHTs examined across the studies were heterogeneous, spanning multiple domains of breast cancer care. Most of the studies focused on clinical decision support systems and decision support interventions.^20–22^^,33,34,41^ Artificial intelligence (AI)-supported screening tools^26–28^^,32^ and tools for assessing patient-reported outcomes and self-reporting^30,35,37,39^ were also reviewed. Three studies covered the field of telemedicine and remote care^23,36,40^ and one study addressed eHealth in general.^29^ Three studies focused on patient data sharing.^25,31,38^ A short description of each DHT can be found in Table S3 of the Supplemental material I.

### Risk of bias for individual studies

Due to the methodological heterogeneity of the included studies, a direct comparison of the risk of bias across the included studies is not feasible. The risk of bias in the individual studies is displayed in the Supplemental material II. Overall, the methodological quality of the included studies was moderate. Most studies had clearly defined aims and appropriate methodological approaches. Several studies exhibited methodological limitations, particularly in relation to sampling and recruitment procedures. Further limitations included low or unreported response rates and incomplete descriptions of analytical procedures. Ethical aspects were consistently addressed, and the methods applied were generally appropriate for answering the respective research questions. All studies were retained to ensure a comprehensive synthesis of the available evidence.

### Data synthesis

In total, 223 text segments were coded, yielding 74 advantage-related codes, 40 disadvantage-related codes, 54 facilitators and 55 barriers. The reviewers coded the data independently, line by line. In most cases, they assigned identical or semantically equivalent codes, or one reviewer's assessment was adopted without further discussion. In 28 cases disagreements had to be discussed to reach a consensus, most commonly concerning whether a specific outcome had been reported by HCPs. Table 2 provides an overview of all analytical themes and their frequency. In Table 3 an overview, linking the different types of DHTs with the corresponding advantages, disadvantages, barriers and facilitators can be found. The coded material for each article, including the coding framework and sample quotations, is available as a MAXQDA file in the Supplemental material III.

**Table 2.** Analytical themes gathered by thematic synthesis.

Advantages			
		**Code count**	**Article count**
Information utilisation		**7**	
	Easier information access	1	1
	Provide additional information	3	2
	Provides useful information	1	1
	Presents information in a useful format	1	1
	Standardised collection of PROs	1	1
Clinical decision-making		**19**	
	Identify areas of concern	1	1
	Early intervention possible	1	1
	Confirm knowledge of patient's problems	2	2
	Improved detection	1	1
	Consistency in screen-reading	1	1
	Can increase medical knowledge	1	1
	Early detection of symptoms	1	1
	Useful to inform clinical judgement	11	8
Economic and practical aspects		**5**	
	Would help effectiveness	1	1
	Improved profitability	3	1
	Economic benefits	1	1
Clinical efficiency and workload management		**11**	
	Supports brevity and action	1	1
	Informing patient via website is time-saving	1	1
	Counteracts staff shortage	1	1
	Easing screen-reading workload	1	1
	Streamlining of routine care	4	2
	Decrease workload	1	1
	Allows quick documentation	2	2
Patient management and care		**32**	
	Information as discussion basis	7	3
	Information given to patient	6	4
	Decrease load on patients	1	1
	Useful to discuss sensitive topics	1	1
	Patient empowerment	5	4
	Contribute to patient management	3	2
	Enhance patient care	9	5

Disadvantages			
		Code count	Article count
Clinical accuracy and interpretation		**13**	
	Loss of detailed information due to standardised data collection	1	1
	Fear of medical risks	1	1
	Just from scores it is not clear whether there is a need on the side of the patient	1	1
	No new information provided	1	1
	Less reliable	2	1
	Harder to see a physical problem	2	1
	Difficult to interpret	1	1
	Risk of automation bias	1	1
	Potential errors	3	2
Quality of care		**10**	
	Loss of ability to tailor information and communication	1	1
	Non-verbal cues are more difficult	1	1
	Lack of ‘human element’	8	4
Data and privacy issues		**1**	
	Information shared with too many parties	1	1
Workflow and working conditions		**11**	
	Formalisation of former ‘analogue’ processes	1	1
	Fear of deterioration of working conditions	1	1
	Fear of loss of competence due to a lack of training	2	1
	Takes more or too much time	7	4
Patient inclusivity and engagement		**5**	
	A lot to take in for patients	1	1
	Concern of poorer support for patients	3	2
	Only applicable for a subset of patients (with technological skills)	1	1

Facilitators			
		Code count	Article count
Workload management		**7**	
	Little interference with clinical routine	4	4
	Automated prompting for double-reading	1	1
	Time saving in comparison to ‘conventional’ tasks	2	1
Quality of care		**4**	
	Second opinion consultations	1	1
	Information on patients’ chemotherapy cycle	1	1
	High sensitivity	2	2
Provider characteristics		**11**	
	Radiology: lower recall rate	1	1
	Junior physician	1	1
	Younger age	1	1
	Owning a smartphone (vs. not owning)	1	1
	Radiology: positive view of computer-aided decision systems	1	1
	Personal organisation	3	3
	Already had positive experiences	2	2
	Personal interest	1	1
Contextual factors		**6**	
	Early-stage cancer treatment	1	1
	Lower percentage of work spent on DHT	1	1
	Hub-hospital physician	1	1
	Medium patient volume (in comparison to low patient volume)	1	1
	Hospital physician (vs. out-patient physician)	1	1
	Academic setting	1	1
Development and implementation strategies		**8**	
	Use of DHT at the end of process	1	1
	Already established routine for e-mail reminders	1	1
	User-involvement in development	1	1
	Recommended by HCPs	2	1
	Leaders involved in implementation	1	1
	Detailed training	2	2
Usability, design and content		**18**	
	Evidence-based	2	2
	Information also available analogous (e.g. printable)	2	2
	Information on interpretation of displayed data	1	1
	Option to comment on task	1	1
	Graphical display of data	3	3
	Appropriate presentation of information	1	1
	Workflow customisable	1	1
	Easy to use	2	2
	Innovation and novelty	2	1
	Risk predictions that incorporated both imaging data and clinical information	1	1
	Objective figures	1	1
	Generalisability	1	1

Barriers			
		Code count	Article count
Data and information management		**2**	
	Unnecessary data collection	1	1
	No human involvement	1	1
Provider characteristics		**3**	
	Cancer specialty = surgery (in comparison to medical oncology)	1	1
	Slow adoption	1	1
	Not willing to inform patient about DHT	1	1
Patient-related barriers		**9**	
	Too much information for patients	3	2
	Information provided too harsh	4	2
	Irregular use by patients	2	2
Organisation and resources		**18**	
	Unclear responsibilities	1	1
	Integration into clinical routine	3	2
	Lack of technical infrastructure	4	2
	Staff shortages	1	1
	Limited time resources	6	5
	Staff changes	1	1
	Insufficient training	2	1
Clinical benefit concerns		**7**	
	Uncertainty about compatibility with existing care	1	1
	Misleading graphical depiction	3	2
	Shorter risk prediction period	1	1
	Automated screen recommendations	1	1
	No accuracy gains over established methods	1	1
Legal and ethical aspects		**6**	
	Concern, that DHT can replace HCPs	4	2
	Uncertainties regarding legal and ethical aspects	2	1
Usability		**10**	
	Technical issues	2	2
	Lack of validation of data	6	5
	Performance not good enough (yet)	1	1
	Log on to more than one site	1	1

All included articles were coded by two researchers each using the line-by-line method, meaning that first codes close to the text were assigned. The descriptive themes were grouped into analytical themes in the domains of advantages and disadvantages as well as barriers and facilitators. An example of the method: the text segment ‘the lack of infrastructure, e.g. computers and printers within the clinics, and the need for internet access were all cited as barriers’^21^ was coded into the descriptive theme ‘lack of technical infrastructure’. This together with similar themes was then used to derive the analytical theme ‘Barriers regarding organisation and resources’. The frequencies shown in the column code count indicate how often the initial code was assigned. A code may have been assigned multiple times per article. The column article count describes the number of articles in which the code was assigned at least once.

**Table 3.** Matrix of DHTs and the corresponding categories.

Focus of DHT	AI screening tool	Clinical decision support system/DESI	Focus on patient data sharing	eHealth in general	Patient-reported outcomes/self-reporting	Telemedicine
No. of included articles	*n* = 4	*n* = 6	*n* = 3	*n* = 1	*n* = 4	*n* = 3
**Categories and code counts**						
Advantages						
Information utilisation	0	0	3	0	4	0
Clinical decision-making	6	5	1	0	7	0
Economic and practical aspects	4	0	1	0	0	0
Clinical efficiency and workload management	3	3	2	0	1	2
Patient management and care	1	15	2	1	12	1
Disadvantages						
Clinical accuracy and interpretation	4	3	1	0	3	2
Quality of care	0	1	3	0	1	5
Data and privacy issues	0	0	1	0	0	0
Workflow and working conditions	5	0	1	0	4	1
Patient inclusivity and engagement	0	3	0	0	1	1
Facilitators						
Workload management	4	1	1	0	1	0
Quality of care	2	0	0	0	1	1
Provider characteristics	2	1	3	3	1	1
Contextual factors	2	0	0	1	0	3
Development and implementation strategies	1	5	0	0	2	0
Usability, design and content	2	7	3	0	6	0
Barriers						
Data and information management	1	0	1	0	0	0
Provider characteristics	0	1	0	0	1	1
Patient-related barriers	0	7	0	0	2	0
Organisation and resources	0	9	2	0	7	0
Clinical benefit concerns	3	2	0	0	2	0
Legal and ethical aspects	3	3	0	0	0	0
Usability	3	3	1	0	3	0

DHT: digital health technology; AI: Artificial intelligence; DESI: decision support intervention.

### Advantages from the perspective of HCPs

The themes derived from the included studies highlight several advantages of DHTs from the perspective of HCPs, particularly in the areas of information use, clinical decision-making, economic and practical aspects, workload management and patient care.

*Patient management and care*: Digital solutions provide a basis for discussions between HCPs and patients,^21,37,41^ and can support the process of providing comprehensive and understandable health information for patients.^21,25,30,41^ They also help to empower patients,^21,29,35,41^ reduce the burden on patients^41^ and facilitate the discussion of sensitive topics.^25^ HCPs reported that DHTs can contribute to patient management^30,37^ and eventually enhance patient care.^28,30,34–37^

*Information utilisation*: DHTs were perceived to enhance information handling by improving access to relevant data,^31^ offering supplementary insights,^35,37^ and presenting information in structured, user-friendly formats.^38^ In addition, standardised collection of patient-reported outcomes ensures consistent patient monitoring and improves data reliability.^35^

*Clinical decision-making*: In terms of clinical decision-making, the results from the included studies suggest that digital tools can help identify problem areas,^35^ enabling early symptom recognition and intervention.^30^ HCPs reported that DHTs can help validate their knowledge of patients’ conditions,^37,39^ ensure more consistent screening results^28^ and contribute to an overall increase in medical expertise.^34^ In seven studies^21,25,26,30,32,34,39^ it has been reported in a more general way that DHTs can be useful as an aid to clinical judgement.

*Economic and practical aspects*: HCPs reported economic benefits^28^ as well as an improved profitability.^32^ When asked to assess the usability of a DHT, HCPs answered that the integration of DHTs would help their effectiveness.^38^

*Clinical efficiency and workload management*: One article reported that HCPs ‘made the system work’ because it supported precise and actionable workflows,^31^ while others highlighted aspects such as saving time by enabling patient communication via digital platforms.^41^ In one article, HCPs reported a reduction in workload regarding screen-reading, helping to counteract staff shortages.^28^ In general, HCPs see an advantage in the ability to document patient data quickly,^31,35^ with DHTs eventually streamlining routine care.^36,41^

### Disadvantages from the perspective of HCPs

In addition to the aforementioned advantages of DHTs, HCPs have expressed several disadvantages that could affect their clinical utility and integration into practice.

*Clinical accuracy and interpretation*: HCPs express concerns about medical risks, for example, an increase in false-positives^28^ and the potential for automation bias,^20^ as digital tools may introduce errors or misinterpretations.^20,28,30^ Standardised data collection can lead to a loss of detailed patient information,^31^ making it more difficult to assess physical conditions accurately.^36^ Additionally, relying solely on automated scores does not always clarify whether a patient has a genuine need for intervention.^39^ In one article, HCPs noted that DHTs fail to provide new insights and only confirm what was already known.^35^

*Quality of care*: In four of the included articles, HCPs highlight the lack of a ‘human element'^23,31,36,39^ as a disadvantage of using DHTs, with HCPs describing DHTs as taking their attention away from the patient.^31^ Furthermore, HCPs expressed, that they felt ‘patronised’ as DHTs cannot replace tailored communication and care to the individual patient.^22^ In addition, non-verbal cues are more difficult to understand when using DHTs.^23^

*Data and privacy issues*: Disadvantages regarding the extent to which sensitive patient information is shared beyond its intended audience by using the DHT were mentioned in one article. There was a reluctance to make confidential notes widely accessible, even within the clinical team, as this could compromise patient privacy and confidentiality.^31^

*Workflow and working conditions*: Deterioration of working conditions was reported as a disadvantage,^28^ for example through the formalisation of previously flexible analogue processes.^23^ In an article on breast cancer screening, HCPs expressed concerns about a decline in professional competence due to inadequate training when DHTs assist with certain tasks.^28^ The added complexity of DHTs may also lead to inefficiencies rather than improvements in workflow.^25,28,30,32^

*Patient inclusivity and engagement*: HCPs were concerned about poorer support,^22,36^ particularly in relation to certain patient groups, as DHTs may cater to a subset of users who are comfortable with digital tools.^22^ A disadvantage was also that DHTs may overwhelm patients in terms of the amount of information presented.^30^

### Facilitators from the perspective of HCPs

Analysis of the included studies identified factors that facilitate the adoption of DHTs by HCPs. These factors span several areas, including individual provider characteristics, contextual influences, strategic implementation approaches, workflow optimisation, quality of care improvements and system usability.

*Development and implementation strategies*: When DHTs are introduced through structured implementation strategies, such as involving users in their development,^33^ using existing workflows (e.g. email reminders)^39^ and integrating them at the end of clinical processes,^27^ HCPs are more likely to accept them. Leadership support^33^ and comprehensive training^21,39^ were also identified as facilitators.

*Usability, design and content*: When DHTs are perceived as user-friendly,^34,38^ evidence-based^20,21^ and innovative,^30^ their adoption increases. Features such as graphical displays,^25,35,39^ clear guidance on data interpretation,^39^ the option to comment on tasks^37^ and customisable workflows^27^ serve as facilitators, making DHTs more accessible and practical.

*Workload management*: HCPs report, that they are more likely to use DHTs, if they have little inference with clinical routines^25,27,30,33^ and automate time-consuming tasks, for example, automated prompting for double-reading.^27^ Their potential to streamline workflows and save time compared to conventional tasks supports implementation.^28^

*Quality of care*: Adoption of DHTs is facilitated if DHTs are perceived as further enhancing quality of care, for example, by facilitating second-opinion consultations through telemedicine^36^ or increasing diagnostic accuracy in radiology.^27^ In one article, HCPs commented that improving a DHT by adding information about the chemotherapy cycle related to the reported symptoms would be helpful.^37^

*Provider characteristics*: HCPs who are younger,^29^ have prior positive experiences with digital tools^23,31^ or have a general interest in technology^29,31^ are more likely to view DHTs positively. Three articles mentioned HCPs personal organisation as a facilitator.^21,31,39^

*Contextual factors*: Settings such as early-stage cancer treatment,^36^ (hub) hospitals^29,40^ and academic institutions^27^ provide favourable conditions for adoption. Additionally, when HCPs spend a moderate portion of their workload on DHTs, they may be more open to their benefits.^32^

### Barriers from the perspective of HCPs

Several barriers hinder the widespread adoption and effective use of DHTs in clinical practice. These barriers include provider characteristics, patient-related challenges, organisational and resource constraints, concerns about clinical utility, legal and ethical issues, usability issues and data management.

*Provider characteristics*: The reluctance to inform patients about DHTs,^22^ and a slow adoption^39^ are barriers when integrating DHTs into clinical practice. One article reports that in certain medical specialties, such as surgery, there is logically less patient contact via telemedicine compared to areas such as medical oncology.^40^

*Patient-related barriers*: HCPs report that they are concerned that patients may be overwhelmed by the amount of information provided in/by DHTs, especially if the information provided is perceived as too harsh.^21,22^ Furthermore, irregular use of DHTs by patients was mentioned as a barrier to implementation.^37,39^

*Organisational and resource limitations*: Issues such as limited time resources,^21,22,30,31,39^ staff shortages,^30^ staff turnover^21^ and insufficient training^30^ are barriers from the perspective of HCPs. Unclear responsibilities within teams,^39^ the integration into established clinical routines^22,39^ and insufficient technical infrastructure^21,22^ were further mentioned as organisational and resource limitations.

*Clinical benefit concerns*: HCPs report concerns regarding the clinical benefits of DHTs as a barrier to adoption. These concerns include uncertainties about their compatibility with existing care processes^22^ and issues such as misleading graphical depictions of data. In the context of AI risk prediction models, automated screening recommendations, shorter risk prediction periods and no accuracy gains over established methods reduced the probability of uptake.^27^

*Legal and ethical aspects*: In two articles, HCPs mentioned the concern about the potential of DHTs to replace HCPs,^22,28^ raising questions about job security and ethical implications.^28^

*Usability*: The lack of validation of data was the most frequently mentioned barrier in the context of the usability of DHTs.^20,21,26,28,39^ Additionally, technical issues in general,^21,31^ poor performance^28^ and the need to log onto more than one site^39^ discourage HCPs from fully adopting digital solutions in their practice.

*Data and information management*: The collection of unnecessary data^31^ and the absence of human involvement^26^ are further barriers mentioned in the included articles.

## Discussion

The aim of this systematic review was to explore and summarise the perceived advantages, disadvantages, barriers and facilitators associated with the adoption of DHTs by HCPs in the context of breast cancer care. A total of 21 studies were included and analysed using thematic synthesis. The findings show a complex interplay of factors that shape HCPs’ perceptions. HCPs evaluate DHTs based on their impact on their professional practice and work environment.

In line with previous literature on DHTs,^1^ HCPs highlighted the potential of DHTs to enhance the quality of care, for example, by improving communication, optimising workflows and supporting clinical decision-making. These benefits are evident in areas such as decision support systems, telemedicine and digital tools for patient-reported outcomes. In our review, HCPs perceived DHTs as valuable for the early detection of symptoms, as a basis for discussion with patients, and as a contribution to better patient management. These findings are consistent with broader evidence that DHTs improve patient self-efficacy and coping skills.^2,8,9,42,43^ Mentioned facilitators in the included studies were positive experiences with technology, which increase motivation and adaptability. Furthermore, as it has been shown before,^44^ a supportive work environment can promote acceptance. However, it should be noted that some of the reported advantages are presented without a detailed example of potential process improvements. For instance, in the category of economic and practical aspects, specific mechanisms through which economic benefits or an improved profitability and effectiveness might be achieved are often not explained. Rather, they reflect HCPs’ perceptions that such advantages could potentially occur.

Although HCPs report that DHTs have the potential to improve clinical workflows, they also mention several disadvantages. A key concern is the added complexity, which reflects previously described challenges relating to the potential increase in mental workload.^45^ This issue may be exacerbated when DHTs fail to provide new insights and only confirm what was already known.^35^ A lack of organisational support (e.g. insufficient training, unclear responsibilities and resource constraints) can hinder widespread adoption. Contextual differences between healthcare settings also appear to shape DHT use and acceptance. One included study found that physicians working in hospital or university settings showed a greater preference for support and side effect documentation via the internet than physicians in outpatient practices.^46^ Similarly, another study reported that patient contacts with breast cancer oncologists in a hub hospital were more than twice as likely to occur via telemedicine than in regional facilities.^40^ One possible explanation could be that academic and clinical environments may be more conducive to the implementation of DHTs due to their infrastructure and institutional support. Previous reviews in the context of breast cancer have shown that regular and competency-based training is essential for effective and ethical use of technology^44^ and targeted interventions have the potential to encourage mHealth prescriptions.^47^

With regards to patient-related factors, HCPs acknowledged the view that DHTs can have both advantages and disadvantages. On the one hand, in the included studies HCPs mention that digital tools can empower patients by providing access to health information, improve communication with HCPs and facilitate informed decisions, as it has also been shown in reviews focusing on the patient perspective.^8,9^ On the other hand, previous literature suggests that DHTs may pose challenges in terms of patient participation and inclusion.^48,49^ Moreover, DHTs may exacerbate social health inequalities if they fail to address the needs of disadvantaged populations, particularly by not ensuring effective access to digital technologies.^50^ Patient characteristics may indirectly influence the utility and adoption of DHTs by HCPs, as some patients may benefit less from these technologies, which can reduce the perceived advantages from the HCP perspective. For example, patients with comorbidities or complex treatment regimens may require more tailored DHTs to meet their needs. Similarly, patients with low digital literacy often face challenges in using digital tools effectively, further limiting the perceived benefit for HCPs. Studies included in this review show, that some HCPs fear that DHTs may overwhelm patients with too much information or fail to meet the needs of people who are less digitally literate.

There are also concerns that digital tools lack a ‘human element’, which can be particularly important in sensitive conversations, such as those related to breast cancer diagnosis and treatment. Due to the emotional nature of interactions in oncology for patients, HCPs express doubts that digital tools could adequately replace personal contact, especially in contexts where non-verbal cues and empathetic responses are essential. A study analysing the perspectives of older women with early breast cancer on telemedicine during post-primary treatment found that patients preferred in-person visits early in care and viewed telemedicine as ‘a little mechanical’ until a strong relationship had been established between them and their clinician.^51^

From the provider perspective, digital tools offer benefits, such as improved efficiency, support for clinical decision-making and better data visualisation, but only when designed and integrated thoughtfully. The lack of validation of data was the most frequently cited concern, along with issues such as technical problems, poor system performance and the need to log into multiple platforms. In addition to the heterogeneity in digital competencies among HCPs this reinforces the need for regular, tailored and competency-based training, which has previously been mentioned.^44^ Overall, our review reveals similar barriers and facilitators in the area of breast cancer as those previously reported in general healthcare.^4^ However, training and education programmes as well as incentives were rarely addressed in the studies we included. This may indicate that these barriers are considered less important than other aspects of breast cancer care, or that they are not reported as often.

A research gap identified in this review is that most of the included studies assess DHTs within the timeframe of defined interventions, focusing on outcomes such as feasibility or usability, but do not evaluate whether the DHTs can be sustained in routine clinical practice over time. Studies, that address long-term use, examine broader usage (e.g. the use of telemedicine in general) rather than specific interventions. However, we included one feasibility trial,^35^ which was followed by a two-round quality improvement study,^39^ providing some insight into long-term integration. HCPs, involved in the quality improvement study, reported positive feedback, but also raised concerns about the potential impact on their workflow. This reflects a common theme across the included studies: although DHTs are generally well received, workflow-related issues can hinder their integration into routine care.

### Strengths and limitations

To our knowledge, this is the first systematic review to identify and summarise perceived benefits and disadvantages for HCPs when implementing DHTs in breast cancer care. We included a sufficient number of studies to address the research question. To enhance trustworthiness, the study selection as well as the analytic process was documented extensively, and decisions were iteratively discussed among reviewers. To ensure a nuanced interpretation of the results, deviant cases were also taken into account in the synthesis. This review synthesises a broad range of studies covering multiple dimensions relevant to the adoption of DHTs, including provider characteristics, patient-related factors and technological considerations.

Regarding the included studies, it should be noted that most of them originated from high-income regions such as the USA, the UK and Western Europe. This limits the generalisability of the findings to other regions and cultural contexts. Moreover, some of the included studies are relatively old, and the digital health landscape has evolved significantly. However, insufficient technical infrastructure was mentioned as a barrier in both 2011^22^ and 2021,^21^ suggesting persistent issues. Another limitation may be a reporting bias towards positive outcomes and advantages. Furthermore, some studies reported HCPs’ perspectives together with patient perspectives, making it difficult to distinguish the specific attitudes and experiences of HCPs. A methodological limitation of this review is that our search was restricted to three databases, and only up to February 2024. These restrictions may have resulted in the exclusion of some relevant recent studies, reporting the assessment of novel DHTs or emerging trends. In particular, given the rapid developments in AI, it is possible that new insights into the potential clinical benefits have emerged since our literature search. This is particularly relevant as HCPs mentioned uncertainties regarding the lack of data validation as a barrier to using DHTs. This may be even more pronounced in AI-based applications.

### Practical and research implications

To facilitate successful implementation DHTs should be developed in collaboration with HCPs to guarantee alignment with clinical workflows, ease of use and technical reliability. Special attention should be given to vulnerable patient groups, such as older people, individuals with low digital literacy and diverse socio-cultural populations, to avoid health inequalities and ensure equitable access. Healthcare facilities should offer structured, competency-based training and carefully integrate digital tools into routine workflows, while also clarifying responsibilities, to facilitate sustainable adoption. From a research perspective, long-term evaluations are needed to assess the sustainability of implementation. Future research should address the long-term impact of DHTs on the quality of care, patient engagement and system-level outcomes.

## Conclusion

This systematic review highlights a number of technological, organisational and individual factors that can influence the acceptance of DHTs by HCPs. While usability and technical performance are important enabling factors, persistent barriers such as lack of validation, technical issues and inadequate integration into clinical workflows might hinder widespread adoption. HCPs should be involved in development processes to address a perceived lack of ‘human element’ and ensure alignment with clinical requirements.

## Supplemental Material

sj-docx-1-dhj-10.1177_20552076251404497 - Supplemental material for Perceived benefits and disadvantages for healthcare professionals when implementing digital health technologies in breast cancer care: A systematic review Supplemental material, sj-docx-1-dhj-10.1177_20552076251404497 for Perceived benefits and disadvantages for healthcare professionals when implementing digital health technologies in breast cancer care: A systematic review by Julia Wendel, Anna-Lena Hofmann, Jonas Widmann, Achim Wöckel, Peter Heuschmann and Jens-Peter Reese in DIGITAL HEALTH

sj-pdf-2-dhj-10.1177_20552076251404497 - Supplemental material for Perceived benefits and disadvantages for healthcare professionals when implementing digital health technologies in breast cancer care: A systematic review Supplemental material, sj-pdf-2-dhj-10.1177_20552076251404497 for Perceived benefits and disadvantages for healthcare professionals when implementing digital health technologies in breast cancer care: A systematic review by Julia Wendel, Anna-Lena Hofmann, Jonas Widmann, Achim Wöckel, Peter Heuschmann and Jens-Peter Reese in DIGITAL HEALTH

sj-mx24-3-dhj-10.1177_20552076251404497 - Supplemental material for Perceived benefits and disadvantages for healthcare professionals when implementing digital health technologies in breast cancer care: A systematic review Supplemental material, sj-mx24-3-dhj-10.1177_20552076251404497 for Perceived benefits and disadvantages for healthcare professionals when implementing digital health technologies in breast cancer care: A systematic review by Julia Wendel, Anna-Lena Hofmann, Jonas Widmann, Achim Wöckel, Peter Heuschmann and Jens-Peter Reese in DIGITAL HEALTH

sj-docx-4-dhj-10.1177_20552076251404497 - Supplemental material for Perceived benefits and disadvantages for healthcare professionals when implementing digital health technologies in breast cancer care: A systematic review Supplemental material, sj-docx-4-dhj-10.1177_20552076251404497 for Perceived benefits and disadvantages for healthcare professionals when implementing digital health technologies in breast cancer care: A systematic review by Julia Wendel, Anna-Lena Hofmann, Jonas Widmann, Achim Wöckel, Peter Heuschmann and Jens-Peter Reese in DIGITAL HEALTH
