# Supplementary material for: Perceived benefits and disadvantages for healthcare professionals when implementing digital health technologies in breast cancer care: A systematic review
Source: Digit Health. 2025 Dec 4;11:20552076251404497. doi: 10.1177/20552076251404497 (PMC12681584; doi:10.1177/20552076251404497)
Supplement: sj-docx-1-dhj-10.1177_20552076251404497 - Supplemental material for Perceived benefits and disadvantages for healthcare professionals when implementing digital health technologies in breast cancer care: A systematic review [file sj-docx-1-dhj-10.1177_20552076251404497.docx]

**Supplementary material for:**

Perceived Benefits and Disadvantages for Healthcare Professionals when Implementing Digital Health Technologies in Breast Cancer Care – A Systematic Review

Wendel, Julia^1,2^; Hofmann, Anna-Lena^1,2^; Widmann, Jonas^1,2^; Wöckel, Achim^3^; Heuschmann, Peter^1,2,4^; Reese, Jens-Peter^1,5^

^1^ University of Würzburg, Institute for Clinical Epidemiology and Biometry, Würzburg, Germany

^2^ University Hospital Würzburg (UKW), Institute for medical Data Science, Würzburg, Germany

^3^ University Hospital Würzburg (UKW), Department of Gynecology and Obstetrics, Würzburg, Germany

^4^ University Hospital Würzburg (UKW), Clinical Trial Centre, Würzburg, Germany

^5^ Technische Hochschule Mittelhessen, University of Applied Sciences, Gießen, Germany

* Correspondence: julia.wendel@uni-wuerzburg.de

*Supplement Table 1. Search strategy.*

| **PubMed:** |
| --- |
| "Physicians" [Mesh] OR "nurse" [Mesh]  AND  "breast cancer" [Mesh]  AND  "Medical Informatics Applications"[Mesh] OR "Digital Health"[Mesh] OR "Digital Technology"[Mesh] OR "Electronic Health Records"[Mesh] OR digital OR online OR internet OR eHealth OR mobile health OR mHealth OR mhealth OR mobile phone OR telemedicine OR website OR patient portal OR web portal OR interface OR health technolog* OR digital intervention OR digital health technolog* |
| "process evaluation"  AND  "breast cancer" [Mesh]  AND  "Medical Informatics Applications"[Mesh] OR "Digital Health"[Mesh] OR "Digital Technology"[Mesh] OR "Electronic Health Records"[Mesh] OR digital OR online OR internet OR eHealth OR mobile health OR mHealth OR mhealth OR mobile phone OR telemedicine OR website OR patient portal OR web portal OR interface OR health technolog* OR digital intervention OR digital health technolog* |
| **ProQuest (PsycInfo):** |
| (physician OR nurse) AND (Medical Informatics Applications OR Digital Health OR Digital Technology OR Electronic Health Records OR digital OR online OR internet OR eHealth OR mobile health OR mHealth OR mhealth OR mobile phone OR telemedicine OR website OR patient portal OR web portal OR interface OR health technology OR digital intervention OR digital health technology) AND breast cancer |
| **IEEE Xplore:** |
| (("physician" OR "nurse" OR "health care provider")  AND  "breast cancer"  AND  ("Medical Informatics Applications" OR "Digital Health" OR "Digital Technology" OR "Electronic Health Records" OR "digital" OR "online" OR "internet" OR "eHealth" OR "mobile health" OR "mHealth" OR "mhealth" OR "mobile phone" OR "telemedicine" OR "website" OR "patient portal" OR "web portal" OR "interface" OR "health technology" OR "digital intervention" OR "digital health technology")) |

*Supplement Table 2. Studies excluded from the review after full text screening.*

| **Database Search** |  |
| --- | --- |
| **Study** | **Reason for exclusion** |
| Ahlstedt Karlsson S, et al. *Person-centred support programme (RESPECT intervention) for women with breast cancer treated with endocrine therapy: a feasibility study.* BMJ Open. 2022;12(10). | no digital intervention |
| Benard VB, et al. *Cancer screening practices among physicians in the National Breast and Cervical Cancer Early Detection Program.* Journal of Women's Health. 2011;20(10):1479-84. | no digital intervention |
| Chee W, et al. *A culturally tailored internet cancer support group for Asian American breast cancer survivors: A randomized controlled pilot intervention study.* Journal of Telemedicine and Telecare. 2017;23(6):618-26. | wrong outcome |
| Emery J, et al. *Computer support for recording and interpreting family histories of breast and ovarian cancer in primary care (RAGs): qualitative evaluation with simulated patients*. Bmj. 1999;319(7201):32-6. | no digital intervention |
| Flávia Oliveira de Almeida Marques da C, et al. *Mobile App (AMOR Mama) for Women With Breast Cancer Undergoing Radiation Therapy: Functionality and Usability Study.* Journal of Medical Internet Research. 2021. | wrong outcome |
| Gao Y, et al. *Digital Breast Tomosynthesis Practice Patterns Following 2011 FDA Approval: A Survey of Breast Imaging Radiologists.* Acad Radiol. 2017;24(8):947-53. | wrong outcome |
| Garvelink MM, et al. *Development of a decision aid about fertility preservation for women with breast cancer in the Netherlands.* Journal of Psychosomatic Obstetrics & Gynecology. 2013;34(4):170-8. | wrong outcome |
| Grimsbø GH, et al. *Cancer patients’ expressions of emotional cues and concerns and oncology nurses’ responses, in an online patient–nurse communication service.* Patient Education and Counseling. 2012;88(1):36-43. | wrong outcome |
| Hahn EE, et al. *Provider perceptions and expectations of breast cancer posttreatment care: a University of California Athena Breast Health Network project*. J Cancer Surviv. 2013;7(3):323-30. | no digital intervention |
| Hill-Kayser CE, et al. *An Internet tool for creation of cancer survivorship care plans for survivors and health care providers: Design, implementation, use and user satisfaction.* Journal of Medical Internet Research. 2009;11(3):1-12. | wrong outcome |
| Jiwa M, et al. *The management of acute adverse effects of breast cancer treatment in general practice: a video-vignette study*. J Med Internet Res. 2014;16(9):e204. | no digital intervention |
| Johnson-Turbes A, et al. *Evaluation of a Web-Based Program for African American Young Breast Cancer Survivors*. Am J Prev Med. 2015;49(6 Suppl 5):S543-9. | wrong population |
| Jones T, et al. *Qualitative analysis of shared decision-making for chemoprevention in the primary care setting: provider-related barriers.* BMC Med Inform Decis Mak. 2022;22(1):208. | wrong outcome |
| Kotranza A, et al. *Virtual Human + Tangible Interface = Mixed Reality Human An Initial Exploration with a Virtual Breast Exam Patient.* 2008 IEEE Virtual Reality Conference; 2008 8-12 March 2008. | no digital intervention |
| Laidsaar-Powell R, et al. *Improving breast cancer nurses' management of challenging situations involving family carers:* *Pilot evaluation of a brief targeted online education module (TRIO-Conflict).* Patient Educ Couns. 2021;104(12):3023-31. | no digital intervention |
| Mouillet G, et al. *Feasibility of health‑related quality of life (HRQoL) assessment for cancer patients using electronic patient‑reported outcome (ePRO) in daily clinical practice.* Quality of Life Research: An International Journal of Quality of Life Aspects of Treatment, Care & Rehabilitation. 2021;30(11):3255-66. | wrong outcome |
| Savelberg W, et al. *Implementing a breast cancer patient decision aid: Process evaluation using medical files and the patients' perspective.* Eur J Cancer Care (Engl). 2021;30(4):e13387. | wrong outcome |
| Schubbe D, et al. *Implementation and sustainability factors of two early-stage breast cancer conversation aids in diverse practices.* Implementation Science. 2021;16:1-14. | no digital intervention |
| Stangl S, et al. *Development and proof-of-concept of a multicenter, patient-centered cancer registry for breast cancer patients with metastatic disease—the “Breast cancer care for patients with metastatic disease” (BRE-4-MED) registry.* Pilot and Feasibility Studies. 2020;6:1. | wrong outcome |
| Taba ST, et al. *Social networks and expertise development for Australian breast radiologists.* BMC Health Serv Res. 2017;17(1):131. | no digital intervention |
| Wheelock AE, et al. *SIS.NET: A randomized controlled trial evaluating a web‐based system for symptom management after treatment of breast cancer.* Cancer. 2015;121(6):893-9. | wrong outcome |
| Whitehead L, et al. *Evaluation of a Remote Symptom Assessment and Management (SAM) System for People Receiving Adjuvant Chemotherapy for Breast or Colorectal Cancer: Mixed Methods Study.* JMIR Cancer. 2020;6(2). | wrong outcome |
| **Citation Search** |  |
| **Study** | **Reason for exclusion** |
| Vashitz G, et al. *Defining and measuring physicians' responses to clinical reminders*. J Biomed Inform. 2009 Apr;42(2):317-26. | wrong outcome |
| Joseph-Williams N, et al. *Implementing shared decision making in the NHS: lessons from the MAGIC programme*. BMJ. 2017 Apr 18;357:j1744. | no digital intervention |
| Graham, I.D. et al. *Physicians' intentions and use of three patient decision aids.* BMC Med Inform Decis Mak. 2007; 7, 20. | no digital intervention |
| Dickinson, R. et al. *Using technology to deliver cancer follow-up: a systematic review.* BMC Cancer. 2014;14, 311. | wrong outcome |
| Petkus, H. et al. *What do senior physicians think about AI and clinical decision support systems: Quantitative and qualitative analysis of data from specialty societies*. Clinical Medicine. 2020; 20, 3. | wrong population |
| Carter, S et al. *The ethical, legal and social implications of using artificial intelligence systems in breast cancer care*. The Breast. 2020. 49. | wrong outcome |
| Reumkens K, et al. *Exploring the preferences of involved health professionals regarding the implementation of an online decision aid to support couples during reproductive decision-making in hereditary cancer: a mixed methods approach*. Fam Cancer. 2019 Apr;18(2):285-291. | wrong population |
| Kelley M, et al. *Oncologists' Perceptions of a Digital Tool to Improve Cancer Survivors' Cardiovascular Health*. ACI open. 2019 Jul;3(2):e78-e87. | wrong population |

*Supplement Table 3. Study characteristics. (* The description of these aspects is related to the assessment of the outcome that is relevant for the review.)*

| **Study** | **Country** | **Methodology*** | **Study Design*** | **Population and Sample Size*** | **Setting (in breast cancer care)** | **Description of digital health technology** | **Outcome** | **Q1 (Advantages and Disadvantages)** | **Q2**  **(Barriers and Facilitators)** |
| --- | --- | --- | --- | --- | --- | --- | --- | --- | --- |
| Bouaud et al, 2015 | France | quantitative | randomized controlled trial | 394 decisions made by multidisciplinary meeting physicians in 3 hospitals of the intervention arm | breast cancer management | OncoDoc 2: a guideline-based CDSS (clinical decision support systems) for breast cancer management | reactance and automation bias of physicians in multidisciplinary meetings when receiving advice of a guideline-based clinical decision support system | no | yes |
| Burton et al, 2021 | UK | mixed-methods | quantitative usage data (e.g. website login) and semi-structured interviews with clinicians | interviews with ten clinicians from six intervention sites (eight surgeons, one oncologist, one nurse practitioner) | breast cancer treatment | two decision support interventions (DESIs), each comprised an online risk prediction model, brief decision aid and an information booklet | Implementation and usage of DESIs and barriers and facilitators to future implementation | yes | yes |
| Caldon et al, 2011 | UK | qualitative | semi-structured interviews | 24 specialist breast clinicians, 21 NHS breast team clinicians, three national opinion leaders | breast cancer treatment | web-based provision of decision support interventions (DESIs) | perceived challenges to the implementation of a DESI into clinical settings | yes | yes |
| Fielding et al, 2005 | UK | qualitative | semi-structured interviews including the assessment of the group behaviour inventory | 24 participants with the following roles (medical, surgical, nursing or allied health professionals, research) from four different hospitals | breast cancer treatment | use of videoconferencing in breast cancer management (multidisciplinary team (MDT) meetings) | attitudes to future use of videoconferencing systems for MDT meetings | yes | yes |
| Garvelink et al, 2012 | Netherlands | mixed-methods | two round Delphi study and additional online focus group | breast cancer patients, breast cancer nurses, oncologists (medical, surgical, and radiotherapy) and gynaecologists specialized in fertility issues | breast cancer treatment | web-based decision aid about fertility preservation | opinions on the implementation of the web-based decision aid | yes | yes |
| Gehrke et al, 2018 | US | qualitative | informal feedback | three nurse navigators with a range of 10 to 17 years of experience as breast cancer oncology nurses | breast cancer aftercare | CSPro-BC app: The app includes a needs assessment and generates a profile of needs. The app provides problem-specific online resources to patients. HCPs have insight in the patient data. | informal feedback on the rationale for the app and clarity and clinical utility of the graphical output | yes | yes |
| Hendrix et al, 2021 | US | quantitative | discrete choice experiment | 91 primary care providers | breast cancer screening | use of artificial intelligence (AI) in breast cancer screening | preferences for attributes of AI in breast cancer screening | no | yes |
| Hendrix et al, 2022 | US | quantitative | discrete choice experiment | 66 radiologists | breast cancer mammography | use of AI during screening mammography interpretation | preferences for attributes of AI in screening mammography interpretation | no | yes |
| Högberg et al, 2023 | Sweden | mixed-methods | observational study (online survey with open and closed questions); triangulation | 47 participants (members of the Swedish Society of Breast Imaging) | mammography screening | use of AI in mammography screening | radiologists' views on the use of AI in mammography screening and their perceptions of risks, benefits and responsibilities as well as the impact on the profession | yes | yes |
| Kirkovits et al, 2016 | Germany | quantitative | Cross-sectional study with a questionnaire-based survey | 120 physicians involved in breast cancer care | breast cancer treatment | eHealth in general, especially future eHealth tools, e.g. a telephone-hotline, which cancer patients could turn to for support as well as future support for patients using the internet or smartphones, and collecting information regarding side effects of therapy via electronic devices | opinions on future eHealth tools and determinants of their use | no | yes |
| Maguire et al, 2008 | UK | mixed-methods | pre-post-design with a combination of semi-structured questionnaires and interviews | convenience sample of 35 nurses who had used the digital intervention | breast cancer treatment | a mobile phone based, advanced symptom management system to monitor and manage chemotherapy-related toxicity within the home care setting. Health professionals at the clinical site can be alerted via a dedicated 24 h pager system, of any incoming symptom reports of concern. | nurses perceptions | yes | yes |
| Oborn et al, 2011 | UK | qualitative | interpretive field study | 28 interviews (16 physicians, 4 nurses, 3 IT administrators, 3 office staff, 2 patients), observation (23 multidisciplinary meetings, 11 other meetings, 19 multidisciplinary clinic sessions), documentation analysis, informal discussions | breast cancer treatment | a web-based clinical information system (SubSys) acting as a shared repository of clinical data on cancer patients | usage of the new system | yes | yes |
| Onega et al, 2010 | US | quantitative | single time point survey | 257 radiologists (all participating in the Breast Cancer Surveillance Consortium) | primary prevention/ screening mammography | computer aided detection (CAD) in screening mammography interpretation | usage of CAD; radiologists perceptions about CAD | yes | yes |
| Raphael et al, 2021 | Netherlands | quantitative | process evaluation within a pre-and post-implementation trial | 13 radiation oncology centres (78 clinicians) with case report forms for 188 patients | breast cancer treatment | An online tool: patient decision aid (PtDA) for decision making on breast cancer radiation therapy. Includes information and textual/ graphical risk communication strategies. Patients can print an overview of their preferences to bring to the consultation with their clinician | factors associated with uptake of PtDA by patients | no | yes |
| Smania, 2016 | US | quantitative | cross-sectional study (survey) | 36 nurse practitioners | breast cancer treatment | a mobile health application to assist decision making and improve knowledge on breast cancer | satisfaction with use of application | yes | no |
| Snyder et al, 2013 | US | mixed-methods | single-arm prospective study (feasibility trial) | eleven clinicians (medical oncologists, nurse practitioners) who managed prostate and breast cancer patients undergoing treatment | breast cancer treatment | "PatientViewpoint": a system which allows clinicians to assign patients PRO (patient-reported outcomes) questionnaires to complete at predefined intervals. The clinicians could access PROs as graphical score reports, and a plain-text table is imported into the electronic health record (EHR). | feasibility of intervention measured by: 1. Usage information from the website 2. Close-ended feedback forms 3. Open-ended interviews | yes | yes |
| Stavrou et al, 2022 | US | quantitative | cross-sectional email survey | 51 breast medical oncology clinicians (physicians (n=36), advance practice practitioners (n=15)) | breast cancer treatment | telemedicine (combined video-audio communications) | perceptions and attitudes towards telemedicine | yes | yes |
| Warrington et al, 2019 | UK | mixed-methods | observational clinical field testing, including brief written feedback, direct observation, documentation, ad hoc verbal feedback | feedback forms: 9 clinical nurse specialists, 5 senior oncologists, 6 oncology trainees  observations: 3 clinical nurse specialists, 2 oncology trainees, 3 senior oncologists | breast cancer treatment and  aftercare | electronic patient self-reporting of adverse events in an online systems accessible for the treating hospital | usability of the online systems | yes | yes |
| Weaver et al, 2021 | US | quantitative | observational study (single time point online survey) | 20 oncology providers (14 physicians, 6 physician assistants/ nurse practitioners) | breast cancer aftercare | the online tool - visualizes data regarding seven modifiable cardiovascular health factors to promote discussions - includes information about receipt of potentially cardiotoxic chemotherapies - was designed to be integrated with electronic health records | usability of the assessment tool among oncology providers | yes | no |
| Wu et al, 2016 | US | qualitative | quality improvement study including cross-sectional, interview-based assessments at two time points | twelve clinicians (5 medical oncologists, 4 radiation oncologists, 3 nurse practitioners) treating breast and prostate cancer patients | breast cancer treatment | *(same intervention as Snyder et al, 2013)* "PatientViewpoint": a system which allows clinicians to assign patients PRO (patient-reported outcomes) questionnaires to complete at predefined intervals. The clinicians could access PROs as graphical score reports, and a plain-text table is imported into the electronic health record (EHR). | usability of the webtool (clinician comments and suggestions for improvement ) | yes | yes |
| Yu et al, 2023 | US | quantitative | retrospective observational study | oncologists (medical oncology, radiation oncology, surgery) from breast cancer cases (n=1535) | patient encounter at a hospital (not further described) | usage of telehealth | odds of an encounter being via telehealth (inclusive of video and audio-only encounters) | no | yes (physician characteristics) |
