# Supplementary material for: Perceived benefits and disadvantages for healthcare professionals when implementing digital health technologies in breast cancer care: A systematic review
Source: Digit Health. 2025 Dec 4;11:20552076251404497. doi: 10.1177/20552076251404497 (PMC12681584; doi:10.1177/20552076251404497)
Supplement: sj-pdf-2-dhj-10.1177_20552076251404497 - Supplemental material for Perceived benefits and disadvantages for healthcare professionals when implementing digital health technologies in breast cancer care: A systematic review [file sj-pdf-2-dhj-10.1177_20552076251404497.pdf]

**Supplementary material for:**

# Perceived Benefits and Disadvantages for Healthcare Professionals when Implementing Digital Health Technologies in Breast Cancer Care – A Systematic Review

Wendel, Julia[1,2]; Hofmann, Anna-Lena[1,2]; Widmann, Jonas[1,2]; Wöckel, Achim[3]; Heuschmann, Peter[1,2,4]; Reese, Jens-Peter[1,5]

[1] University of Würzburg, Institute for Clinical Epidemiology and Biometry, Würzburg, Germany
[2] University Hospital Würzburg (UKW), Institute for medical Data Science, Würzburg, Germany
[3] University Hospital Würzburg (UKW), Department of Gynecology and Obstetrics, Würzburg, Germany
[4] University Hospital Würzburg (UKW), Clinical Trial Centre, Würzburg, Germany
[5] Technische Hochschule Mittelhessen, University of Applied Sciences, Gießen, Germany

* Correspondence: julia.wendel@uni-wuerzburg.de

Risk of Bias Assessment for individual studies

Bouaud et al, 2015; Mixed-methods Appraisal Tool, Items quantitative descriptive

| **Items "quantitative descriptive"** | **yes** | **no** | **cant't tell** |
|---|---|---|---|
| *4.1. Is the sampling strategy relevant to address the research question?* | x | | |
| Comment 4.1:<br>Yes. Collection of all data on decisions. | | | |
| | | | |
| *4.2. Is the sample representative of the target population?* | x | | |
| Comment 4.2:<br>yes. Automated collection of data on decisions made by physicians | | | |
| | | | |
| *4.3. Are the measurements appropriate?* | x | | |
| Comment 4.3:<br>automatically collected information on performed navigation for decision and and comparison with subsequent navigation, which is carried out without knowledge of the initial decision | | | |
| | | | |
| *4.4. Is the risk of nonresponse bias low?* | x | | |
| Comment 4.4:<br>Yes, s. 4.1/4.2. | | | |
| | | | |
| *4.5. Is the statistical analysis appropriate to answer the research question?* | | | x |
| Comment 4.5:<br>Statistical analysis (e.g. software used, handling of missing data) not further described | | | |

Burton et al, 2021; Mixed-methods Appraisal Tool, Items mixed-methods design

| **Items "mixed-methods Design"** | **yes** | **no** | **cant't tell** |
|---|---|---|---|
| *5.1 Is there an adequate rationale for using a mixed methods design to address the research question?* | x | | |
| Comment 5.1:<br>yes. They used a process evaluation with multiple methods, to assess implementation fidelity, analysing quantitative process indicators as well as interview data | | | |
| | | | |
| *5.2. Are the different components of the study effectively integrated to answer the research question?* | x | | |
| Comment 5.2:<br>yes. The data collection methods cover the various dimensions of implementation fidelity (s. table 1) | | | |
| | | | |
| *5.3. Are the outputs of the integration of qualitative and quantitative components adequately interpreted?* | x | | |
| Comment 5.3:<br>The results are combined to create a comprehensive picture. | | | |
| | | | |
| *5.4. Are divergences and inconsistencies between quantitative and qualitative results adequately addressed?* | | | x |
| Comment 5.4:<br>not further described. | | | |
| | | | |
| *5.5. Do the different components of the study adhere to the quality criteria of each tradition of the methods involved?* | x | | |
| Comment 5.5:<br>yes. Limitations resulting from the recruitment strategy are reported. | | | |

Fielding et al, 2005; Mixed-methods Appraisal Tool, Items mixed-methods design

| **Items "mixed-methods Design"** | **yes** | **no** | **cant't tell** |
|---|---|---|---|
| *5.1 Is there an adequate rationale for using a mixed methods design to address the research question?* | x | | |
| Comment 5.1:<br>Group behaviour inventory for quantitative assessment and interviews to collect further data on attitudes | | | |
| | | | |
| *5.2. Are the different components of the study effectively integrated to answer the research question?* | | x | |
| Comment 5.2:<br>No. Group behaviour inventory focuses on standard MDT meeting, no information in videoconferencing attitudes. | | | |
| | | | |
| *5.3. Are the outputs of the integration of qualitative and quantitative components adequately interpreted?* | x | | |
| Comment 5.3:<br>Yes. The results are combined to create a comprehensive picture. | | | |
| | | | |
| *5.4. Are divergences and inconsistencies between quantitative and qualitative results adequately addressed?* | | | x |
| Comment 5.4:<br>No divergencies described. | | | |
| | | | |
| *5.5. Do the different components of the study adhere to the quality criteria of each tradition of the methods involved?* | | | x |
| Comment 5.5:<br>methods for interview not further described | | | |

Garvelink et al, 2012; Mixed-methods Appraisal Tool, Items mixed-methods design

| **Items "mixed-methods Design"** | **yes** | **no** | **cant't tell** |
|---|---|---|---|
| *5.1 Is there an adequate rationale for using a mixed methods design to address the research question?* | x | | |
| Comment 5.1:<br>The paper describes the results of a two round Delphi study and additional online focus group (experts consensus/ no consensus) | | | |
| | | | |
| *5.2. Are the different components of the study effectively integrated to answer the research question?* | x | | |
| Comment 5.2:<br>sequential design with two delphi rounds and subsequent online discussion | | | |
| | | | |
| *5.3. Are the outputs of the integration of qualitative and quantitative components adequately interpreted?* | x | | |
| Comment 5.3:<br>yes and in context with literature | | | |
| | | | |
| *5.4. Are divergences and inconsistencies between quantitative and qualitative results adequately addressed?* | | | x |
| Comment 5.4:<br>no divergencies described | | | |
| | | | |
| *5.5. Do the different components of the study adhere to the quality criteria of each tradition of the methods involved?* | | x | |
| Comment 5.5:<br>participants of delphi study were not heterogenous, but mostly in favour of the intervention | | | |

Hendrix et al, 2021; Mixed-methods Appraisal Tool, Items quantitative descriptive

| **Items "quantitative descriptive"** | **yes** | **no** | **cant't tell** |
|---|---|---|---|
| *4.1. Is the sampling strategy relevant to address the research question?* | x | | |
| Comment 4.1:physicians and nurse practicioners in the US | | | |
| | | | |
| *4.2. Is the sample representative of the target population?* | | | |
| Comment 4.2: in the first step, the sample was randomly selected, however, the majority of respondents were recruited via snowball sampling | | x | |
| | | | |
| *4.3. Are the measurements appropriate?* | x | | |
| Comment 4.3:<br>discrete-choice experiement | | | |
| | | | |
| *4.4. Is the risk of nonresponse bias low?* | | x | |
| Comment 4.4:<br>6% of the invited stakeholders participated (however, a "real" response rate can not be calculated due to the snowball sampling) | | | |
| | | | |
| *4.5. Is the statistical analysis appropriate to answer the research question?* | x | | |
| Comment 4.5: | | | |

Hendrix et al, 2022; Mixed-methods Appraisal Tool, Items quantitative descriptive

| **Items "quantitative descriptive"** | **yes** | **no** | **cant't tell** |
|---|---|---|---|
| *4.1. Is the sampling strategy relevant to address the research question?* | x | | |
| Comment 4.1: radiology practices | | | |
| | | | |
| *4.2. Is the sample representative of the target population?* | | | |
| Comment 4.2: practices were sampled among the authors' professional networks | | x | |
| | | | |
| *4.3. Are the measurements appropriate?* | x | | |
| Comment 4.3:<br>discrete-choice experiement | | | |
| | | | |
| *4.4. Is the risk of nonresponse bias low?* | x | | |
| Comment 4.4:<br>Based on power calculations, the recommended minimum sample size for an analysis without interactions between attributes in this experimental design was 62 respondents -> A total of 66 of 150 invited radiologists participated | | | |
| | | | |
| *4.5. Is the statistical analysis appropriate to answer the research question?* | x | | |
| Comment 4.5:<br>latent-class analysis | | | |

Högberg et al, 2023; Mixed-methods Appraisal Tool, Items mixed-methods design

| **Items "mixed-methods Design"** | **yes** | **no** | **cant't tell** |
|---|---|---|---|
| *5.1 Is there an adequate rationale for using a mixed methods design to address the research question?* | x | | |
| Comment 5.1:<br>qualitative free-text answers to complement the quantitative assessment | | | |
| | | | |
| *5.2. Are the different components of the study effectively integrated to answer the research question?* | x | | |
| Comment 5.2:<br>method triangulation witht quantitative assessment underpinned with qualitative citations | | | |
| | | | |
| *5.3. Are the outputs of the integration of qualitative and quantitative components adequately interpreted?* | x | | |
| Comment 5.3:<br>yes | | | |
| | | | |
| *5.4. Are divergences and inconsistencies between quantitative and qualitative results adequately addressed?* | x | | |
| Comment 5.4:<br>Contradictory opinions are also described; e.g. page 5 section "technological development" | | | |
| | | | |
| *5.5. Do the different components of the study adhere to the quality criteria of each tradition of the methods involved?* | x | | |
| Comment 5.5: | | | |

Kirkovits et al, 2016; Mixed-methods Appraisal Tool, Items quantitative descriptive

| **Items "quantitative descriptive"** | **yes** | **no** | **cant't tell** |
|---|---|---|---|
| *4.1. Is the sampling strategy relevant to address the research question?* | x | | |
| Comment 4.1:<br>sampling of physicians participating in breast cancer care | | | |
| *4.2. Is the sample representative of the target population?* | | x | |
| Comment 4.2:<br>The questionnaire was handed out to participants only on two occasions in 2012 in Munich (COMBATing Breast Cancer conference in Munich, Germany and at a breast cancer-specific meeting organized by Tumor Center Munich) | | | |
| *4.3. Are the measurements appropriate?* | x | | |
| Comment 4.3:<br>The questionnaire invited medical professionals to share their views on future eHealth tools. The first section focused on a proposed telephone hotline that cancer patients could use for support. The second section contained questions about potential future tools for patient support, specifically involving the Internet, smartphone use, and electronic methods for tracking therapy side effects. Responses for this section were rated on a five-point scale, ranging from strong agreement (1) to strong disagreement (5). | | | |
| *4.4. Is the risk of nonresponse bias low?* | x | | |
| Comment 4.4:<br>Out of 154 participants who attended the data collection events, a total of 120 active medical professionals completed the questionnaire | | | |
| *4.5. Is the statistical analysis appropriate to answer the research question?* | x | | |
| Comment 4.5:<br>Student t-tests were applied to analyze differences between groups. Odds ratios were used to describe variations across parameters, and P-values were calculated with a significance level < 5% | | | |

Maguire et al, 2008; Mixed-methods Appraisal Tool, Items mixed-methods design

| **Items "mixed-methods Design"** | **yes** | **no** | **cant't tell** |
|---|---|---|---|
| *5.1 Is there an adequate rationale for using a mixed methods design to address the research question?* | x | | |
| Comment 5.1:<br>quantitative assessment with in-depth interviews | | | |
| | | | |
| *5.2. Are the different components of the study effectively integrated to answer the research question?* | x | | |
| Comment 5.2:<br>semi-structured interviews were conducted with a convenience sample of 10 nurses from the original 35 recruited to the study, who met the inclusion and exclusion criteria as previously described, from six of the seven participating sites. | | | |
| | | | |
| *5.3. Are the outputs of the integration of qualitative and quantitative components adequately interpreted?* | x | | |
| Comment 5.3: | | | |
| | | | |
| *5.4. Are divergences and inconsistencies between quantitative and qualitative results adequately addressed?* | x | | |
| Comment 5.4:<br>e.g. regarding tailored self-care advice (p.385) | | | |
| | | | |
| *5.5. Do the different components of the study adhere to the quality criteria of each tradition of the methods involved?* | x | | |
| Comment 5.5:<br>yes, however the findings from the perception questionnaires and interviews may not fully represent the entire sample for two reasons. First, not all nurses who completed the pre-study questionnaire also completed the post-study questionnaire, and vice versa. Additionally, although 10 nurses who had used ASyMS were interviewed, participants were selected using convenience sampling | | | |

Onega et al, 2010; Mixed-methods Appraisal Tool, Items quantitative descriptive

| **Items "quantitative descriptive"** | **yes** | **no** | **cant't tell** |
|---|---|---|---|
| *4.1. Is the sampling strategy relevant to address the research question?* | x | | |
| Comment 4.1:<br>Radiologists who interpreted mammograms at any of the facilities contributing data to the seven mammography registries were invited to participate in a mailed survey | | | |
| | | | |
| *4.2. Is the sample representative of the target population?* | x | | |
| Comment 4.2:<br>relatively high response rate (71%) | | | |
| | | | |
| *4.3. Are the measurements appropriate?* | | | x |
| Comment 4.3:<br>Seven predefined statements with fixed response categories had to be rated. It is not reported whether respondents were given the opportunity to add further perceptions. | | | |
| | | | |
| *4.4. Is the risk of nonresponse bias low?* | x | | |
| Comment 4.4:<br>relatively high response rate | | | |
| | | | |
| *4.5. Is the statistical analysis appropriate to answer the research question?* | x | | |
| Comment 4.5:<br>latent class analysis | | | |

Raphael et al, 2021; Mixed-methods Appraisal Tool, Items quantitative descriptive

| **Items "quantitative descriptive"** | **yes** | **no** | **cant't tell** |
|---|---|---|---|
| *4.1. Is the sampling strategy relevant to address the research question?* | x | | |
| Comment 4.1:<br>patients who received an invitation for the use of the patient-decision aid where invited to participate in the study | | | |
| | | | |
| *4.2. Is the sample representative of the target population?* | | | x |
| Comment 4.2:<br>not known how many patients were eligible for the study but didn't get invited or refused to participate | | | |
| | | | |
| *4.3. Are the measurements appropriate?* | | x | |
| Comment 4.3:<br>The authors wanted to report patient, clinician, and organizational factors that are related to the uptake of the intervention. They assess a relatively small and predefinied amount of factors. It is not clear, wether there are different contextual factors. | | | |
| | | | |
| *4.4. Is the risk of nonresponse bias low?* | | | x |
| Comment 4.4:<br>s. 4.2 | | | |
| | | | |
| *4.5. Is the statistical analysis appropriate to answer the research question?* | x | | |
| Comment 4.5: | | | |

Smania, 2015; Mixed-methods Appraisal Tool, Items quantitative descriptive

| **Items "quantitative descriptive"** | **yes** | **no** | **cant't tell** |
|---|---|---|---|
| *4.1. Is the sampling strategy relevant to address the research question?* | | x | |
| Comment 4.1:<br>only attendees of a specific conference. High risk of selection bias | | | |
| | | | |
| *4.2. Is the sample representative of the target population?* | | x | |
| Comment 4.2:<br>s. 4.1 | | | |
| | | | |
| *4.3. Are the measurements appropriate?* | | | x |
| Comment 4.3:<br>methods and results are described rudimentary | | | |
| | | | |
| *4.4. Is the risk of nonresponse bias low?* | x | | |
| Comment 4.4:<br>Conference participants were invited to take part in the survey in advance and on site. However, attention should be drawn to points 4.1 and 4.2. | | | |
| | | | x |
| *4.5. Is the statistical analysis appropriate to answer the research question?* | | | |
| Comment 4.5:<br>methods and results are described rudimentary | | | |

Snyder et al, 2013; Mixed-methods Appraisal Tool, Items mixed-methods design

| **Items "mixed-methods Design"** | **yes** | **no** | **cant't tell** |
|---|---|---|---|
| *5.1 Is there an adequate rationale for using a mixed methods design to address the research question?* | x | | |
| Comment 5.1:<br>quantitative assessment with in-depth interviews | | | |
| | | | |
| *5.2. Are the different components of the study effectively integrated to answer the research question?* | x | | |
| Comment 5.2:<br>decription of both components and reporting of both | | | |
| | | | |
| *5.3. Are the outputs of the integration of qualitative and quantitative components adequately interpreted?* | x | | |
| Comment 5.3:<br>results report both as well as contrary results | | | |
| | | | |
| *5.4. Are divergences and inconsistencies between quantitative and qualitative results adequately addressed?* | | | x |
| Comment 5.4:<br>no divergences or inconsistencies reported | | | |
| | | | |
| *5.5. Do the different components of the study adhere to the quality criteria of each tradition of the methods involved?* | x | | |
| Comment 5.5: | | | |

Stavrou et al, 2022; Mixed-methods Appraisal Tool, Items quantitative descriptive

| **Items "quantitative descriptive"** | **yes** | **no** | **cant't tell** |
|---|---|---|---|
| *4.1. Is the sampling strategy relevant to address the research question?* | x | | |
| Comment 4.1: | | | |
| | | | |
| *4.2. Is the sample representative of the target population?* | | x | |
| Comment 4.2:<br>the sample is relatively small and selective | | | |
| | | | |
| *4.3. Are the measurements appropriate?* | x | | |
| Comment 4.3:<br>various advantages and disadvantages assessed | | | |
| | | | |
| *4.4. Is the risk of nonresponse bias low?* | | x | |
| Comment 4.4:<br>response rate of 72% | | | |
| | | | |
| *4.5. Is the statistical analysis appropriate to answer the research question?* | x | | |
| Comment 4.5:<br>descriptive as well as Fishers exact test for comparison of groups | | | |

Warrington et al, 2019; Mixed-methods Appraisal Tool, Items mixed-methods design

| **Items "mixed-methods Design"** | **yes** | **no** | **cant't tell** |
|---|---|---|---|
| *5.1 Is there an adequate rationale for using a mixed methods design to address the research question?* | x | | |
| Comment 5.1:<br>broader understanding of usability analysing descriptive data on frequency of usage as well as written and verbal feedback | | | |
| | | | |
| *5.2. Are the different components of the study effectively integrated to answer the research question?* | x | | |
| Comment 5.2:<br>yes, although the results for the clinicians are only described briefly | | | |
| | | | |
| *5.3. Are the outputs of the integration of qualitative and quantitative components adequately interpreted?* | x | | |
| Comment 5.3: | | | |
| | | | |
| *5.4. Are divergences and inconsistencies between quantitative and qualitative results adequately addressed?* | x | | |
| Comment 5.4:<br>usage patterns and suggestions for change are described broadly | | | |
| | | | |
| *5.5. Do the different components of the study adhere to the quality criteria of each tradition of the methods involved?* | | | x |
| Comment 5.5:<br>methods not described in detail | | | |

Weaver et al, 2021; Mixed-methods Appraisal Tool, Items quantitative descriptive

| **Items "quantitative descriptive"** | **yes** | **no** | **cant't tell** |
|---|---|---|---|
| *4.1. Is the sampling strategy relevant to address the research question?* | | | |
| Comment 4.1:<br>yes, physicians as well as physician assistants/nurse practitioners were recruited | x | | |
| | | | |
| *4.2. Is the sample representative of the target population?* | | | |
| Comment 4.2:<br>sample size of n=20 was driven by feasibility concerns | | | x |
| | | | |
| *4.3. Are the measurements appropriate?* | | | |
| Comment 4.3:<br>Provider usability was assessed using 6 questions utilized in our previous study of general internal medicine physicians | x | | |
| | | | |
| *4.4. Is the risk of nonresponse bias low?* | | | |
| Comment 4.4:<br>sample size was driven by feasibility concerns | | x | |
| | | | |
| *4.5. Is the statistical analysis appropriate to answer the research question?* | | | |
| Comment 4.5:<br>yes, descriptive analysis of likert-scaled items | x | | |

Yu et al, 2023; Mixed-methods Appraisal Tool, Items quantitative descriptive

| **Items "quantitative descriptive"** | **yes** | **no** | **cant't tell** |
|---|---|---|---|
| *4.1. Is the sampling strategy relevant to address the research question?* | | | |
| Comment 4.1:<br>The objective of this study was to examine telehealth encounters for patients diagnosed with breast, colorectal, and lung cancer within the Dartmouth Health system, home to a rural National Cancer Institute comprehensive cancer center. We first sought to identify characteristics of oncologists associated with telehealth encounters post COVID-19. | x | | |
| | | | |
| *4.2. Is the sample representative of the target population?* | x | | |
| Comment 4.2:<br>Patients were identified from the institutional tumor registry and linked to the EPIC electronic health records at Dartmouth Health to identify their clinical encounters from 3 months prior to 12 months following their cancer diagnosis or through September 2021, whichever came first. | | | |
| | | | |
| *4.3. Are the measurements appropriate?* | | | |
| Comment 4.3:<br>Multilevel models of the odds of an encounter being via telehealth by cancer type post COVID-19. | x | | |
| | | | |
| *4.4. Is the risk of nonresponse bias low?* | x | | |
| Comment 4.4: | | | |
| | | | |
| *4.5. Is the statistical analysis appropriate to answer the research question?* | x | | |
| Comment 4.5: | | | |

www.casp-uk.net
info@casp-uk.net
Summertown Pavilion, Middle Way Oxford OX2 7LG

CASP Checklist: 10 questions to help you make sense of a Qualitative research

How to use this appraisal tool: Three broad issues need to be considered when appraising a qualitative study:

- Are the results of the study valid? (Section A)
- What are the results? (Section B)
- Will the results help locally? (Section C)

The 10 questions on the following pages are designed to help you think about these issues systematically. The first two questions are screening questions and can be answered quickly. If the answer to both is "yes", it is worth proceeding with the remaining questions. There is some degree of overlap between the questions, you are asked to record a "yes", "no" or "can't tell" to most of the questions. A number of italicised prompts are given after each question. These are designed to remind you why the question is important. Record your reasons for your answers in the spaces provided.

About: These checklists were designed to be used as educational pedagogic tools, as part of a workshop setting, therefore we do not suggest a scoring system. The core CASP checklists (randomised controlled trial & systematic review) were based on JAMA 'Users' guides to the medical literature 1994 (adapted from Guyatt GH, Sackett DL, and Cook DJ), and piloted with health care practitioners.

For each new checklist, a group of experts were assembled to develop and pilot the checklist and the workshop format with which it would be used. Over the years overall adjustments have been made to the format, but a recent survey of checklist users reiterated that the basic format continues to be useful and appropriate.

Referencing: we recommend using the Harvard style citation, i.e.: *Critical Appraisal Skills Programme (2018). CASP (insert name of checklist i.e. Qualitative) Checklist. [online] Available at: URL. Accessed: Date Accessed.*

©CASP this work is licensed under the Creative Commons Attribution – Non-Commercial-Share A like. To view a copy of this license, visit http://creativecommons.org/licenses/by-nc-sa/3.0/ www.casp-uk.net

CASP Critical Appraisal Skills Programme

Paper for appraisal and reference: Gehrke_2018

| Section A: Are the results valid? |
|---|

1. Was there a clear statement of the aims of the research?

Yes ✔
Can't Tell ☐
No ☐

HINT: Consider
- what was the goal of the research
- why it was thought important
- its relevance

Comments: Experience of app use by participating healthcare professionals

2. Is a qualitative methodology appropriate?

Yes ✔
Can't Tell ☐
No ☐

HINT: Consider
- If the research seeks to interpret or illuminate the actions and/or subjective experiences of research participants
- Is qualitative research the right methodology for addressing the research goal

Comments:

| Is it worth continuing? |
|---|

3. Was the research design appropriate to address the aims of the research?

Yes ☐
Can't Tell ☐
No ✔

HINT: Consider
- if the researcher has justified the research design (e.g. have they discussed how they decided which method to use)

Comments: Documentation of informal feedback can provide initial information about the attitudes of healthcare professionals and describe implementation mechanisms, but cannot replace a qualified evaluation

4. Was the recruitment strategy appropriate to the aims of the research?

| | |
|---|---|
| Yes | ☐ |
| Can't Tell | ☐ |
| No | ☑ |

HINT: Consider
- If the researcher has explained how the participants were selected
- If they explained why the participants they selected were the most appropriate to provide access to the type of knowledge sought by the study
- If there are any discussions around recruitment (e.g. why some people chose not to take part)

Comments: highly selective sample; small sample size

5. Was the data collected in a way that addressed the research issue?

| | |
|---|---|
| Yes | ☑ |
| Can't Tell | ☐ |
| No | ☐ |

HINT: Consider
- If the setting for the data collection was justified
- If it is clear how data were collected (e.g. focus group, semi-structured interview etc.)
- If the researcher has justified the methods chosen
- If the researcher has made the methods explicit (e.g. for interview method, is there an indication of how interviews are conducted, or did they use a topic guide)
- If methods were modified during the study. If so, has the researcher explained how and why
- If the form of data is clear (e.g. tape recordings, video material, notes etc.)
- If the researcher has discussed saturation of data

Comments:

CASP
Critical Appraisal
Skills Programme

6. Has the relationship between researcher and participants been adequately considered?

| | |
|---|---|
| Yes | |
| Can't Tell | ✔ |
| No | |

HINT: Consider

- If the researcher critically examined their own role, potential bias and influence during (a) formulation of the research questions (b) data collection, including sample recruitment and choice of location
- How the researcher responded to events during the study and whether they considered the implications of any changes in the research design

Comments:

Section B: What are the results?

7. Have ethical issues been taken into consideration?

| | |
|---|---|
| Yes | ✔ |
| Can't Tell | |
| No | |

HINT: Consider

- If there are sufficient details of how the research was explained to participants for the reader to assess whether ethical standards were maintained
- If the researcher has discussed issues raised by the study (e.g. issues around informed consent or confidentiality or how they have handled the effects of the study on the participants during and after the study)
- If approval has been sought from the ethics committee

Comments: yes, ethical approval obtained

CASP
Critical Appraisal
Skills Programme

8. Was the data analysis sufficiently rigorous?

| | |
|---|---|
| Yes | ✔ |
| Can't Tell | |
| No | |

HINT: Consider
- If there is an in-depth description of the analysis process
- If thematic analysis is used. If so, is it clear how the categories/themes were derived from the data
- Whether the researcher explains how the data presented were selected from the original sample to demonstrate the analysis process
- If sufficient data are presented to support the findings
- To what extent contradictory data are taken into account
- Whether the researcher critically examined their own role, potential bias and influence during analysis and selection of data for presentation

Comments: informal feedback is comprehensively described

9. Is there a clear statement of findings?

| | |
|---|---|
| Yes | ✔ |
| Can't Tell | |
| No | |

HINT: Consider whether
- If the findings are explicit
- If there is adequate discussion of the evidence both for and against the researcher's arguments
- If the researcher has discussed the credibility of their findings (e.g. triangulation, respondent validation, more than one analyst)
- If the findings are discussed in relation to the original research question

Comments:

Critical Appraisal Skills Programme

Section C: Will the results help locally?

10. How valuable is the research?

HINT: Consider
- If the researcher discusses the contribution the study makes to existing knowledge or understanding (e.g. do they consider the findings in relation to current practice or policy, or relevant research-based literature
- If they identify new areas where research is necessary
- If the researchers have discussed whether or how the findings can be transferred to other populations or considered other ways the research may be used

Comments: Results were considered during further app development

CASP
Critical Appraisal
Skills Programme

www.casp-uk.net
info@casp-uk.net
Summertown Pavilion, Middle
Way Oxford OX2 7LG

CASP Checklist: 10 questions to help you make sense of a Qualitative research

How to use this appraisal tool: Three broad issues need to be considered when appraising a qualitative study:

- Are the results of the study valid? (Section A)
- What are the results? (Section B)
- Will the results help locally? (Section C)

The 10 questions on the following pages are designed to help you think about these issues systematically. The first two questions are screening questions and can be answered quickly. If the answer to both is "yes", it is worth proceeding with the remaining questions. There is some degree of overlap between the questions, you are asked to record a "yes", "no" or "can't tell" to most of the questions. A number of italicised prompts are given after each question. These are designed to remind you why the question is important. Record your reasons for your answers in the spaces provided.

About: These checklists were designed to be used as educational pedagogic tools, as part of a workshop setting, therefore we do not suggest a scoring system. The core CASP checklists (randomised controlled trial & systematic review) were based on JAMA 'Users' guides to the medical literature 1994 (adapted from Guyatt GH, Sackett DL, and Cook DJ), and piloted with health care practitioners.

For each new checklist, a group of experts were assembled to develop and pilot the checklist and the workshop format with which it would be used. Over the years overall adjustments have been made to the format, but a recent survey of checklist users reiterated that the basic format continues to be useful and appropriate.

Referencing: we recommend using the Harvard style citation, i.e.: *Critical Appraisal Skills Programme (2018). CASP (insert name of checklist i.e. Qualitative) Checklist. [online] Available at: URL. Accessed: Date Accessed.*

©CASP this work is licensed under the Creative Commons Attribution – Non-Commercial-Share A like. To view a copy of this license, visit http://creativecommons.org/licenses/by-nc-sa/3.0/ www.casp-uk.net

CASP
Critical Appraisal
Skills Programme

Paper for appraisal and reference: Oborn 2011

## Section A: Are the results valid?

1. Was there a clear statement of the aims of the research?

| Option | |
|---|---|
| Yes | ✔ |
| Can't Tell | |
| No | |

HINT: Consider
- what was the goal of the research
- why it was thought important
- its relevance

Comments:

2. Is a qualitative methodology appropriate?

| Option | |
|---|---|
| Yes | ✔ |
| Can't Tell | |
| No | |

HINT: Consider
- If the research seeks to interpret or illuminate the actions and/or subjective experiences of research participants
- Is qualitative research the right methodology for addressing the research goal

Comments: using data from observations and documents supports the findings from the interviews and informal discussions and allows a broader insight

## Is it worth continuing?

3. Was the research design appropriate to address the aims of the research?

| Option | |
|---|---|
| Yes | ✔ |
| Can't Tell | |
| No | |

HINT: Consider
- if the researcher has justified the research design (e.g. have they discussed how they decided which method to use)

Comments: broad research approach to fully understand implementation and usage processes

4. Was the recruitment strategy appropriate to the aims of the research?

| | |
|---|---|
| Yes | |
| Can't Tell | ✔ |
| No | |

HINT: Consider
- If the researcher has explained how the participants were selected
- If they explained why the participants they selected were the most appropriate to provide access to the type of knowledge sought by the study
- If there are any discussions around recruitment (e.g. why some people chose not to take part)

Comments: not reported in detail (e.g. non-respondance not described)

5. Was the data collected in a way that addressed the research issue?

| | |
|---|---|
| Yes | ✔ |
| Can't Tell | |
| No | |

HINT: Consider
- If the setting for the data collection was justified
- If it is clear how data were collected (e.g. focus group, semi-structured interview etc.)
- If the researcher has justified the methods chosen
- If the researcher has made the methods explicit (e.g. for interview method, is there an indication of how interviews are conducted, or did they use a topic guide)
- If methods were modified during the study. If so, has the researcher explained how and why
- If the form of data is clear (e.g. tape recordings, video material, notes etc.)
- If the researcher has discussed saturation of data

Comments: extensive data collection using mutliple qualitative methods (Interviews, informal discussions, observations, documentation analysis)

CASP Critical Appraisal Skills Programme

6. Has the relationship between researcher and participants been adequately considered?

Yes ☐
Can't Tell ☑
No ☐

HINT: Consider

- If the researcher critically examined their own role, potential bias and influence during (a) formulation of the research questions (b) data collection, including sample recruitment and choice of location
- How the researcher responded to events during the study and whether they considered the implications of any changes in the research design

Comments: not described

Section B: What are the results?

7. Have ethical issues been taken into consideration?

Yes ☑
Can't Tell ☐
No ☐

HINT: Consider

- If there are sufficient details of how the research was explained to participants for the reader to assess whether ethical standards were maintained
- If the researcher has discussed issues raised by the study (e.g. issues around informed consent or confidentiality or how they have handled the effects of the study on the participants during and after the study)
- If approval has been sought from the ethics committee

Comments: The first author obtained access and appropriate ethics approval as part of a broader research project to study multidisciplinary collaboration in cancer services

8. Was the data analysis sufficiently rigorous?

Yes ✔
Can't Tell ☐
No ☐

HINT: Consider
- If there is an in-depth description of the analysis process
- If thematic analysis is used. If so, is it clear how the categories/themes were derived from the data
- Whether the researcher explains how the data presented were selected from the original sample to demonstrate the analysis process
- If sufficient data are presented to support the findings
- To what extent contradictory data are taken into account
- Whether the researcher critically examined their own role, potential bias and influence during analysis and selection of data for presentation

Comments:

9. Is there a clear statement of findings?

Yes ✔
Can't Tell ☐
No ☐

HINT: Consider whether
- If the findings are explicit
- If there is adequate discussion of the evidence both for and against the researcher's arguments
- If the researcher has discussed the credibility of their findings (e.g. triangulation, respondent validation, more than one analyst)
- If the findings are discussed in relation to the original research question

Comments:

Critical Appraisal Skills Programme

Section C: Will the results help locally?

10. How valuable is the research?

HINT: Consider
- If the researcher discusses the contribution the study makes to existing knowledge or understanding (e.g. do they consider the findings in relation to current practice or policy, or relevant research-based literature
- If they identify new areas where research is necessary
- If the researchers have discussed whether or how the findings can be transferred to other populations or considered other ways the research may be used

Comments: detailed description of usage patterns showing many varieties between non-use and use/adoption
in parts very specific

CASP
Critical Appraisal Skills Programme

www.casp-uk.net
info@casp-uk.net
Summertown Pavilion, Middle Way Oxford OX2 7LG

CASP Checklist: 10 questions to help you make sense of a Qualitative research

How to use this appraisal tool: Three broad issues need to be considered when appraising a qualitative study:

- Are the results of the study valid? (Section A)
- What are the results? (Section B)
- Will the results help locally? (Section C)

The 10 questions on the following pages are designed to help you think about these issues systematically. The first two questions are screening questions and can be answered quickly. If the answer to both is "yes", it is worth proceeding with the remaining questions. There is some degree of overlap between the questions, you are asked to record a "yes", "no" or "can't tell" to most of the questions. A number of italicised prompts are given after each question. These are designed to remind you why the question is important. Record your reasons for your answers in the spaces provided.

About: These checklists were designed to be used as educational pedagogic tools, as part of a workshop setting, therefore we do not suggest a scoring system. The core CASP checklists (randomised controlled trial & systematic review) were based on JAMA 'Users' guides to the medical literature 1994 (adapted from Guyatt GH, Sackett DL, and Cook DJ), and piloted with health care practitioners.

For each new checklist, a group of experts were assembled to develop and pilot the checklist and the workshop format with which it would be used. Over the years overall adjustments have been made to the format, but a recent survey of checklist users reiterated that the basic format continues to be useful and appropriate.

Referencing: we recommend using the Harvard style citation, i.e.: *Critical Appraisal Skills Programme (2018). CASP (insert name of checklist i.e. Qualitative) Checklist. [online] Available at: URL. Accessed: Date Accessed.*

©CASP this work is licensed under the Creative Commons Attribution – Non-Commercial-Share A like. To view a copy of this license, visit http://creativecommons.org/licenses/by-nc-sa/3.0/ www.casp-uk.net

Paper for appraisal and reference: Wu et al, 2016

## Section A: Are the results valid?

| | | |
|---|---|---|
| 1. Was there a clear statement of the aims of the research? | Yes ✔<br>Can't Tell ☐<br>No ☐ | HINT: Consider<br>• what was the goal of the research<br>• why it was thought important<br>• its relevance |

Comments: this study focused specifically on the usability of PatientViewpoint (a webtool) in a subgroup of purposively sampled patients and clinicians

| | | |
|---|---|---|
| 2. Is a qualitative methodology appropriate? | Yes ✔<br>Can't Tell ☐<br>No ☐ | HINT: Consider<br>• If the research seeks to interpret or illuminate the actions and/or subjective experiences of research participants<br>• Is qualitative research the right methodology for addressing the research goal |

Comments: There were two quality improvement rounds, each of which included a cross-sectional, interview based assessment.

## Is it worth continuing?

| | | |
|---|---|---|
| 3. Was the research design appropriate to address the aims of the research? | Yes ✔<br>Can't Tell ☐<br>No ☐ | HINT: Consider<br>• if the researcher has justified the research design (e.g. have they discussed how they decided which method to use) |

Comments: This was not a qualitative study in that the data were not collected with the goal of achieving thematic saturation, but rather to inform adjustments.

Critical Appraisal Skills Programme

4. Was the recruitment strategy appropriate to the aims of the research?

| | |
|---|---|
| Yes | ✔ |
| Can't Tell | |
| No | |

HINT: Consider
- If the researcher has explained how the participants were selected
- If they explained why the participants they selected were the most appropriate to provide access to the type of knowledge sought by the study
- If there are any discussions around recruitment (e.g. why some people chose not to take part)

Comments: Twelve of 13 clinicians (92 %) approached agreed to participate in the study (5 medical oncologists, 4 radiation oncologists, 3 nurse practitioners; 8 were male and 4 were female)

5. Was the data collected in a way that addressed the research issue?

| | |
|---|---|
| Yes | ✔ |
| Can't Tell | |
| No | |

HINT: Consider
- If the setting for the data collection was justified
- If it is clear how data were collected (e.g. focus group, semi-structured interview etc.)
- If the researcher has justified the methods chosen
- If the researcher has made the methods explicit (e.g. for interview method, is there an indication of how interviews are conducted, or did they use a topic guide)
- If methods were modified during the study. If so, has the researcher explained how and why
- If the form of data is clear (e.g. tape recordings, video material, notes etc.)
- If the researcher has discussed saturation of data

Comments:

6. Has the relationship between researcher and participants been adequately considered?

| | |
|---|---|
| Yes | |
| Can't Tell | ✔ |
| No | |

HINT: Consider
- If the researcher critically examined their own role, potential bias and influence during (a) formulation of the research questions (b) data collection, including sample recruitment and choice of location
- How the researcher responded to events during the study and whether they considered the implications of any changes in the research design

Comments:

Section B: What are the results?

7. Have ethical issues been taken into consideration?

| | |
|---|---|
| Yes | ✔ |
| Can't Tell | |
| No | |

HINT: Consider
- If there are sufficient details of how the research was explained to participants for the reader to assess whether ethical standards were maintained
- If the researcher has discussed issues raised by the study (e.g. issues around informed consent or confidentiality or how they have handled the effects of the study on the participants during and after the study)
- If approval has been sought from the ethics committee

Comments: The Johns Hopkins School of Medicine IRB approved the study, including the current quality improvement component.

CASP
Critical Appraisal
Skills Programme

8. Was the data analysis sufficiently rigorous?

| | |
|---|---|
| Yes | ✔ |
| Can't Tell | |
| No | |

HINT: Consider
- If there is an in-depth description of the analysis process
- If thematic analysis is used. If so, is it clear how the categories/themes were derived from the data
- Whether the researcher explains how the data presented were selected from the original sample to demonstrate the analysis process
- If sufficient data are presented to support the findings
- To what extent contradictory data are taken into account
- Whether the researcher critically examined their own role, potential bias and influence during analysis and selection of data for presentation

Comments: analysed according to the Plan-Do-Study-Act (PDSA) model for quality improvement

9. Is there a clear statement of findings?

| | |
|---|---|
| Yes | ✔ |
| Can't Tell | |
| No | |

HINT: Consider whether
- If the findings are explicit
- If there is adequate discussion of the evidence both for and against the researcher's arguments
- If the researcher has discussed the credibility of their findings (e.g. triangulation, respondent validation, more than one analyst)
- If the findings are discussed in relation to the original research question

Comments: findings are described with supporting citations as well as the presentation of contrasting findings

CASP
Critical Appraisal
Skills Programme

| Section C: Will the results help locally? |
| --- |

10. How valuable is the research?

HINT: Consider
- If the researcher discusses the contribution the study makes to existing knowledge or understanding (e.g. do they consider the findings in relation to current practice or policy, or relevant research-based literature
- If they identify new areas where research is necessary
- If the researchers have discussed whether or how the findings can be transferred to other populations or considered other ways the research may be used

Comments: according to the Plan-Do-Study-Act (PDSA) findings were integrated into the implementation process and several adjustments were made

www.casp-uk.net
info@casp-uk.net
Summertown Pavilion, Middle Way Oxford OX2 7LG

CASP Checklist: 10 questions to help you make sense of a Qualitative research

How to use this appraisal tool: Three broad issues need to be considered when appraising a qualitative study:

- Are the results of the study valid? (Section A)
- What are the results? (Section B)
- Will the results help locally? (Section C)

The 10 questions on the following pages are designed to help you think about these issues systematically. The first two questions are screening questions and can be answered quickly. If the answer to both is "yes", it is worth proceeding with the remaining questions. There is some degree of overlap between the questions, you are asked to record a "yes", "no" or "can't tell" to most of the questions. A number of italicised prompts are given after each question. These are designed to remind you why the question is important. Record your reasons for your answers in the spaces provided.

About: These checklists were designed to be used as educational pedagogic tools, as part of a workshop setting, therefore we do not suggest a scoring system. The core CASP checklists (randomised controlled trial & systematic review) were based on JAMA 'Users' guides to the medical literature 1994 (adapted from Guyatt GH, Sackett DL, and Cook DJ), and piloted with health care practitioners.

For each new checklist, a group of experts were assembled to develop and pilot the checklist and the workshop format with which it would be used. Over the years overall adjustments have been made to the format, but a recent survey of checklist users reiterated that the basic format continues to be useful and appropriate.

Referencing: we recommend using the Harvard style citation, i.e.: *Critical Appraisal Skills Programme (2018). CASP (insert name of checklist i.e. Qualitative) Checklist. [online] Available at: URL. Accessed: Date Accessed.*

©CASP this work is licensed under the Creative Commons Attribution – Non-Commercial-Share A like. To view a copy of this license, visit http://creativecommons.org/licenses/by-nc-sa/3.0/ www.casp-uk.net

Critical Appraisal Skills Programme

Paper for appraisal and reference: Caldon et al, 2011

| Section A: Are the results valid? |
|---|

1. Was there a clear statement of the aims of the research?

Yes ✔
Can't Tell ☐
No ☐

HINT: Consider
- what was the goal of the research
- why it was thought important
- its relevance

Comments: examine specialist breast clinicians' opinions about the provision of a DESI

2. Is a qualitative methodology appropriate?

Yes ✔
Can't Tell ☐
No ☐

HINT: Consider
- If the research seeks to interpret or illuminate the actions and/or subjective experiences of research participants
- Is qualitative research the right methodology for addressing the research goal

Comments: examine opinions

| Is it worth continuing? |
|---|

3. Was the research design appropriate to address the aims of the research?

Yes ✔
Can't Tell ☐
No ☐

HINT: Consider
- if the researcher has justified the research design (e.g. have they discussed how they decided which method to use)

Comments:

CASP Critical Appraisal Skills Programme

| | | | |
|---|---|---|---|
| 4. Was the recruitment strategy appropriate to the aims of the research? | Yes | ✔ | HINT: Consider<br>• If the researcher has explained how the participants were selected<br>• If they explained why the participants they selected were the most appropriate to provide access to the type of knowledge sought by the study<br>• If there are any discussions around recruitment (e.g. why some people chose not to take part) |
| | Can't Tell | | |
| | No | | |

Comments: non-responders described

| | | | |
|---|---|---|---|
| 5. Was the data collected in a way that addressed the research issue? | Yes | ✔ | HINT: Consider<br>• If the setting for the data collection was justified<br>• If it is clear how data were collected (e.g. focus group, semi-structured interview etc.)<br>• If the researcher has justified the methods chosen<br>• If the researcher has made the methods explicit (e.g. for interview method, is there an indication of how interviews are conducted, or did they use a topic guide)<br>• If methods were modified during the study. If so, has the researcher explained how and why<br>• If the form of data is clear (e.g. tape recordings, video material, notes etc.)<br>• If the researcher has discussed saturation of data |
| | Can't Tell | | |
| | No | | |

Comments: data saturation was not discussed

CASP Critical Appraisal Skills Programme

| | | | |
|---|---|---|---|
| 6. Has the relationship between researcher and participants been adequately considered? | Yes | ✔ | HINT: Consider<br>• If the researcher critically examined their own role, potential bias and influence during (a) formulation of the research questions (b) data collection, including sample recruitment and choice of location<br>• How the researcher responded to events during the study and whether they considered the implications of any changes in the research design |
| | Can't Tell | | |
| | No | | |

Comments: non-clinical researchers conducted the interviews

## Section B: What are the results?

| | | | |
|---|---|---|---|
| 7. Have ethical issues been taken into consideration? | Yes | ✔ | HINT: Consider<br>• If there are sufficient details of how the research was explained to participants for the reader to assess whether ethical standards were maintained<br>• If the researcher has discussed issues raised by the study (e.g. issues around informed consent or confidentiality or how they have handled the effects of the study on the participants during and after the study)<br>• If approval has been sought from the ethics committee |
| | Can't Tell | | |
| | No | | |

Comments: ethical approval

CASP Critical Appraisal Skills Programme

8. Was the data analysis sufficiently rigorous?

Yes ☑
Can't Tell ☐
No ☐

HINT: Consider
- If there is an in-depth description of the analysis process
- If thematic analysis is used. If so, is it clear how the categories/themes were derived from the data
- Whether the researcher explains how the data presented were selected from the original sample to demonstrate the analysis process
- If sufficient data are presented to support the findings
- To what extent contradictory data are taken into account
- Whether the researcher critically examined their own role, potential bias and influence during analysis and selection of data for presentation

Comments: five main themes are described in more detail

9. Is there a clear statement of findings?

Yes ☑
Can't Tell ☐
No ☐

HINT: Consider whether
- If the findings are explicit
- If there is adequate discussion of the evidence both for and against the researcher's arguments
- If the researcher has discussed the credibility of their findings (e.g. triangulation, respondent validation, more than one analyst)
- If the findings are discussed in relation to the original research question

Comments:

CASP
Critical Appraisal
Skills Programme

| Section C: Will the results help locally? |
| --- |

10. How valuable is the research?

HINT: Consider
- If the researcher discusses the contribution the study makes to existing knowledge or understanding (e.g. do they consider the findings in relation to current practice or policy, or relevant research-based literature
- If they identify new areas where research is necessary
- If the researchers have discussed whether or how the findings can be transferred to other populations or considered other ways the research may be used

| Comments: findings are discussed extensively |
| --- |
